# Knockdown of PGM1 enhances anticancer effects of orlistat in gastric cancer under glucose deprivation

**DOI:** 10.1186/s12935-021-02193-3

**Published:** 2021-09-10

**Authors:** Bo Cao, Huan Deng, Hao Cui, Ruiyang Zhao, Hanghang Li, Bo Wei, Lin Chen

**Affiliations:** 1 Medical School of Chinese PLA, Beijing, 100853 China; 2 Department of General Surgery, The First Medical Center, Chinese PLA General Hospital, Beijing, 100853 China; 3 School of Medicine, Nankai University, Tianjin, 300071 China

**Keywords:** Gastric cancer, Phosphoglucomutase 1, Orlistat, Glucose deprivation, Cancer metabolism, Apoptosis

## Abstract

**Background:**

Phosphoglucomutase 1 (PGM1) acts as an important regulator in glucose metabolism. However, the role of PGM1 in gastric cancer (GC) remains unclear. This study aims to investigate the role of PGM1 and develop novel regimens based on metabolic reprogramming in GC.

**Methods:**

Correlation and enrichment analyses of PGM1 were conducted based on The Cancer Genome Atlas database. Data derived from the Kaplan–Meier Plotter database were analyzed to evaluate correlations between PGM1 expression and survival time of GC patients. Cell counting kit-8, 5-Ethynyl-2-deoxyuridine, flow cytometry assays, generation of subcutaneous tumor and lung metastasis mouse models were used to determine growth and metastasis in vitro and in vivo. Cell glycolysis was detected by a battery of glycolytic indicators, including lactate, pyruvic acid, ATP production and glucose uptake. Fatty Acid Synthase (FASN) activity and expression levels of lipid enzymes were determined to reflect on lipid metabolism.

**Results:**

Correlation and enrichment analyses suggested that PGM1 was closely associated with cell viability, proliferation and metabolism. PGM1 was overexpressed in GC tissues and cell lines. High PGM1 expression served as an indicator of shorter survival for specific subpopulation of GC patients. It was also correlated with pathological tumor stage and pathological tumor node metastasis stage of GC. Under the glucose deprivation condition, knockdown of PGM1 significantly suppressed cell viability, proliferation and glycolysis, whereas lipid metabolism was enhanced. Orlistat, as a drug that was designed to inhibit FASN activity, effectively induced apoptosis and suppressed lipid metabolism in GC. However, orlistat conversely increased glycolytic levels. Orlistat exhibited more significant inhibitive effects on GC progression after knockdown of PGM1 under glucose deprivation due to combination of glycolysis and lipid metabolism both in vitro and in vivo.

**Conclusions:**

Downregulation of PGM1 expression under glucose deprivation enhanced anti-cancer effects of orlistat. This combination application may serve as a novel strategy for GC treatment.

**Supplementary Information:**

The online version contains supplementary material available at 10.1186/s12935-021-02193-3.

## Background

Gastric cancer (GC) is one of the most malignant tumors worldwide. The latest investigation of global cancer statistics showed that there were over 10,00,000 new cases of GC and that GC caused an estimated 7,69,000 deaths in 2020. It ranks fifth in incidence and fourth in mortality worldwide [1]. Despite advancements in surgical techniques and chemotherapeutic regimens, many patients with advanced or late-stage GC still have poor prognosis. How to effectively inhibit GC progression is the hot topic of current medical research. In recent years, targeted drugs have attracted much attention and significantly improved therapeutic regimens. However, the subpopulation who can potentially harvest benefits from targeted drugs is limited and the associated medical costs are relatively high. It is eagerly required to develop drugs that can be widely applied and simultaneously have high efficacy.

Metabolic reprogramming is a hallmark of cancer cells, and can provide sufficient energy and substances to allow adaptation to the highly proliferative state [2]. This universal feature determines that most of cancers can be effectively attenuated by metabolic inhibition. The critical regulators of glycolysis were gradually identified and inhibition of them has been proved as an effective approach of cancer treatment [3]. However, tumors usually survive in the glucose-deprived microenvironment, which is different from the in-vitro cultivation environment. Glucose starvation exerts stress to cancer cells and they actively reprogram glycolysis and alternative pathways to cope with harsh conditions. Metabolic compensation may be an important reason that glycolysis inhibition cannot thoroughly eliminate cancer cells in vivo. Lipids serve as the important energy source during nutrient deprivation. Many rate-limiting enzymes and regulators in lipid metabolism are abnormally expressed and participated in carcinogenesis [4, 5]. Inspired by this cue, researchers investigated roles of current metabolism drugs in cancer suppression. Orlistat, a type of fatty acid synthase (FASN) inhibitor, is preliminarily used for weight loss. Numerous studies revealed its anticancer effects and potential value of clinical therapy [6–8].

Phosphoglucomutase 1 (PGM1) is the enzyme involved in glycogen metabolism. It reversibly converts glucose-1-phosphate to glucose-6-phosphate. The critical role of PGM1 in regulating glucose metabolism and cancer progression was reported [9, 10]. Therefore, it is speculated that PGM1 acts as an oncogenic factor in GC and regulates metabolic reprogramming. In this study, in-vitro and in-vivo experiments were performed to verify our assumptions and develop novel therapeutic regimens to promote translational application of PGM1 knockdown.

## Methods

### Correlation and enrichment analyses

Profiles of gene expression in GC tissues were downloaded from The Cancer Genome Atlas (TCGA) database. Pearson correlation analysis was performed to screen the 600 genes that were most positively or most negatively associated with PGM1 expression, which were then selected for enrichment analysis to identify PGM1 biological functions in GC. Gene ontology (GO) and Kyoto Encyclopedia of Genes and Genomes (KEGG) analyses were conducted based on the clusterProfiler R software package R.

### Clinical specimen

A total of 40 pairs of GC tissues and corresponding adjacent noncancerous tissues were collected from patients who underwent gastrectomy at Chinese PLA General Hospital from March 2020 to October 2020. Pathological examinations were performed by two independent pathologists. This study was approved by the Ethical Committee of Chinese PLA General Hospital (PLAGH-S2017-061-01). Informed consent was obtained from included patients.

### Cell culture

GC cell lines (BGC-823, MKN-28, MGC-803, SGC-7901, HGC-27 and AGS), a human gastric epithelial cell line (GES-1) and a human embryonic kidney cell line (HEK-293 T) were purchased from American Type Culture Collection (MD, USA). BGC-823 cells stably carrying luciferase (luc-BGC-823) were previously established and stored in our laboratory. Cells were cultivated in an incubator at 5% CO_2_ atmosphere and 37 ℃. For the cells that were used for resuscitation, common cultivation, lentivirus packaging and other routine operations, they were grown in high-glucose (25 mM glucose) Dulbecco’s modified Eagle’s medium (DMEM, Thermo Fisher, Waltham, MA, China) supplemented with 10% fetal bovine serum (FBS, Kangyuan Biotech, Tianjin, China) to keep cells in good conditions. Low-glucose (LG) or normal-glucose (NG) medium was used for in-vitro experiments, including cell viability, proliferation, glycolysis and lipid metabolism detection and apoptosis assays as indicated. The LG medium comprised of glucose-free DMEM (Thermo Fisher) supplemented with 10% FBS and 2.5 mM glucose. The NG medium contained glucose-free DMEM, 10% FBS and 10 mM glucose [11].

### shRNA transfection and lentivirus infection

An shRNA against PGM1 was designed and synthesized by JTS Scientific (Wuhan, China). Cell transfection was performed using Lipofectamine 3000 (Thermo Fisher) according to the manufacturer’s protocol. To establish cell lines with stable knockdown of PGM1, a lentiviral packaging kit (Yeasen, Shanghai, China) was used to produce lentivirus media for cell infection. BGC-823 and MKN-28 cells were seeded at the appropriate density and then treated with lentivirus for 24 h. 5 μg/mL puromycin was then added into medium to conduct chemical selection and exclude uninfected cells.

### Quantitative real-time PCR (qRT-PCR) analysis

Total RNA was extracted from clinical specimens using TRIzol Reagent (Invitrogen, NY, USA). ExScript RT-PCR kit (TaKaRa, Japan) was used to perform reverse transcription. Then, cDNA was amplified using SYBR Premix Ex Taq II (TaKaRa) and Archimed X4 system (RocGene, Beijing, China). 2^−ΔΔCt^ method was employed to calculate relative expression of target genes. β-actin served as the internal control gene. The primers for PGM1 were 5′- CGACTCCTTTACGGAACTCA-3′ (forward) and 5′-TCCAGTGGTTTGGCGAAT-3′ (reverse). The primers for β-actin were 5′- TCGTGCGTGACATTAAGGAG-3′ (forward) and 5′- ATGCCAGGGTACATGGTGGT-3′ (reverse).

### Western blot (WB) analysis

For the preparation of WB samples, cells were harvested and lysed in radio immunoprecipitation assay (RIPA) buffer (Solarbio, Beijing, China). Cell debris was removed by centrifugation. Protein quantification was conducted using BCA Protein Assay Kit (Thermo Fisher). SDS loading buffer (Solarbio) was added to the protein supernatants, which were then heated at 100 °C for 15 min. Equal amounts of protein were separated by SDS-PAGE and transferred onto polyvinylidene fluoride membranes (Millipore, MA, USA). The membranes were blocked using 5% skim milk at room temperature for 1 h. The primary and secondary antibodies used in this study were purchased from Abcam (Cambridge, UK). The membranes were incubated with primary antibodies overnight at 4 °C and secondary antibodies at room temperature for 1 h. The blots were imaged using ECL Western Blotting Substrate (Solarbio) and a WB imaging system (Tanon, Beijing, China). The original WB pictures were displayed in Additional file 1.

### Immunohistochemistry (IHC) analysis

Tissue slides were prepared following the methods described in the previous study [12]. The primary and secondary antibodies used in IHC staining were purchased from Abcam. Sections were scanned by Pannoramic Scanning Electron Microscope (3D HISTECH). IHC staining scores were determined using the H-score method. Staining intensity was scored as 0, no staining; 1+ , weak; 2+ , moderate; 3+ , strong.

### Cell counting kit-8 (CCK-8) assay

CCK-8 assay (Abmole Bioscience, Shanghai, China) was used to measure GC viability in vitro. 3 × 10^3^ cells were seeded in 96-well plates and grown in the incubator. At the indicated time, the complete medium used for cell cultivation was removed, and 10% CCK-8 solution diluted by the FBS-free medium was added into wells. The plates were incubated at 37 ℃ for 1 h protected from light. The absorbance of the CCK-8 solution at 450 nm was measured by a microplate reader (Biotek, VT, USA).

### 5-Ethynyl-2-deoxyuridine (EdU) assay

The EdU assay is an approach to measure the proliferative capability of cells. Cell Proliferation EdU Image Kit was purchased from Abbkine (Wuhan, China). The experiments were performed according to the manufacturer’s protocol. The nuclei were stained with 4′,6-diamidino-2-phenylindole (DAPI, Abbkine) to determine the total number of cells. Fluorescent images were observed under the fluorescence microscope. The original EdU pictures were displayed in Additional file 1.

### Metabolic experiments

To investigate glycolysis levels in GC, Lactate Colorimetric Assay Kit II, Pyruvate Colorimetric/Fluorometric Assay Kit, ATP Colorimetric/Fluorometric Assay Kit and Glucose Uptake Colorimetric Assay Kit (Biovision, CA, USA) were used according to the manufacturer’s protocols. Measurement of FASN activity was conducted using FASN Activity Kit (Solarbio). Absorbances were measured using the microplate reader as indicated by the corresponding protocols.

### Cell apoptosis assay

Cells were harvested and washed with cold phosphate-buffered saline (PBS, Solarbio). FITC Annexin V Apoptosis Detection Kit 1 (BD Pharmingen, NJ, USA) was used to stain cells and detect apoptosis induced by orlistat. Apoptotic cells were detected by a FACSort Flow Cytometer (BD Pharmingen). Cells that were Annexin V- and propidium iodide (PI)- positive were regarded as late apoptotic or necrotic cells. Annexin V-positive but PI-negative was the hallmark of early apoptosis. Living cells were negative for both Annexin V and PI.

### Animal experiment

In vivo experiments were conducted to further validate the efficacies of PGM1 knockdown and orlistat treatment. Four-week-old male nude mice were purchased from Charles River (Beijing, China) and housed under specific pathogen-free conditions. The mice were randomly divided with 8 subjects in each group. To generate mouse models with subcutaneous cancer, a total of 5 × 10^6^ luc-BGC-823 cells were subcutaneously injected into the nude mice. The longest and shortest diameters were determined using a vernier caliper every 5 days. Tumor volume = (longest diameter × shortest diameter^2^)/2. For evaluation of metastatic capability, 2 × 10^6^ luc-BGC-823 cells were suspended in PBS and injected into tail veins of nude mice. 1 week after construction of the two models, mice were treated with 240 μg/g of orlistat or the corresponding solvent daily by intraperitoneal injection. After 30 days, the mice were intraperitoneally injected with 1.5 mg D-luciferin (Solarbio) dissolved in PBS and imaged using an in vivo imaging system (PerkinElmer, USA). To achieve euthanasia of the nude mice, they were placed in the chamber without CO_2_ addition. After 5 min of environment adaptation, CO_2_ was injected at the speed of 5 L/min, which was calculated as 20% volume of the chamber per minute. When cardiac arrest of all mice was observed, they were maintained in the chamber for additional 2 min and regarded as dead. The subcutaneous tumors were immediately harvested and used for determination of lactate acid production and FASN activity. The animal experiments were approved by the Ethical Committee of Animal Center of Chinese PLA General Hospital (2018-X10-05).

### Statistical analysis

SPSS 25.0 and Prism 7.0 were used to conduct statistical analysis. The data was presented as means ± standard deviation (SD). The normality of data distribution was confirmed synthetically using Q-Q plot and Shapiro–Wilk test. Two-sided Student’s t test and one-way ANOVA were used to compare variables between groups. Survival analysis was performed by Kaplan–Meier method. Chi-square test was used to examine the correlation between PGM1 expression and clinicopathological characteristics. The experiments were performed least in triplicate unless otherwise indicated. *P* < 0.05 was regarded as the statistical significance.

## Results

### PGM1 expression is negatively correlated with survival outcome of GC patients and overexpressed in GC

**Fig. 1:**
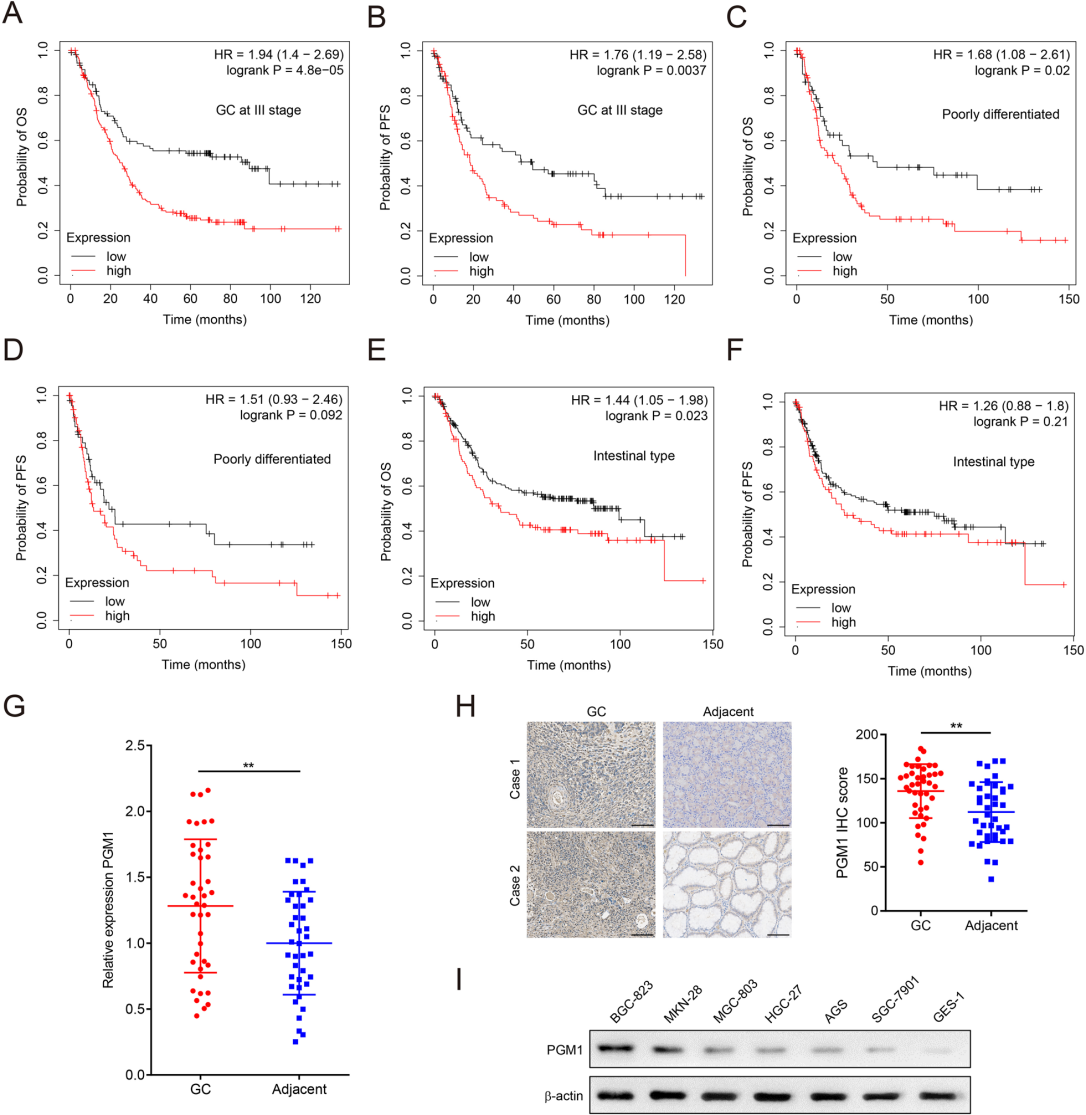
PGM1 expression is negatively correlated with survival outcome of GC patients and overexpressed in GC. **a**, **b** OS (**a**) and PFS (**b**) of GC patients at III stage who were divided into low-PGM1 and high-PGM1 groups according to the Kaplan–Meier plotter database. **c**, **d** OS (**c**) and PFS (**d**) of patients with poorly differentiated GC who were divided into low-PGM1 and high-PGM1 groups according to the Kaplan–Meier plotter database. **e**, **f** OS (**e**) and PFS (**f**) of patients with the intestinal type of GC who were divided into low-PGM1 and high-PGM1 groups according to the Kaplan–Meier plotter database. **g** qRT-PCR analysis to analyze PGM1 mRNA expression in 40 pairs of GC and adjacent noncancerous tissues. **h** IHC analysis to detect PGM1 expression in 40 pairs of GC and adjacent noncancerous tissues. IHC images of two cases are shown on the left and the corresponding diagram of 40 cases are on the right. Scale bar: 100 μm. **i** WB analysis to show the PGM1 expression between GC and gastric epithelial cell lines. Data are shown as the mean ± SD. **P < 0.01. GC: gastric cancer; PGM1: Phosphoglucomutase 1; HR: hazard ratio; OS: overall survival; PFS: progression-free survival; qRT-PCR: quantitative real-time PCR; WB: western blot; IHC: immunohistochemistry; SD: standard deviation

To investigate the role of PGM1 in GC, we analyzed the correlation between PGM1 expression and the survival time of GC patients based on the Kaplan–Meier plotter database (kmplot.com/analysis). We found that patients with GC at stage III (Fig. 1a, b), simultaneously with higher expression of PGM1, had significantly shorter overall survival (OS) and progression-free survival (PFS). Patients with poorly differentiated GC (Fig. 1c) and intestinal type of GC in the PGM1-high group had lower OS (Fig. 1e). However, there were no significant differences of PFS between PGM1-high and PGM1-low groups (Fig. 1d, f). Next, we measured PGM1 mRNA expression in 40 pairs of GC and adjacent noncancerous tissues. The results showed that PGM1 was overexpressed in GC (Fig. 1g). The clinicopathological features were then analyzed and PGM1 expression was correlated with pathological tumor (pT) stage and pathological tumor node metastasis (pTNM) stage of GC patients (Table.1). IHC analysis verified that the expression of PGM1 protein was significantly higher in GC tissues (Fig. 1h). PGM1 expression was also upregulated in GC cell lines, especially in BGC-823 and MKN-28, compared to the gastric epithelial cell line GES-1 (Fig. 1i). Collectively, PGM1 was overexpressed in GC tissues and correlated with pT and pTNM stages. High expression of PGM1 indicated poor prognosis of specific subpopulation of GC patients.

**Table 1:** Correlation between PGM1 expression and clinicopathological characteristics of 40 GC patients

Characteristics	Case number	High (n = 20)	Low (n = 20)	*P* value
Age at surgery (years)				0.749
< 60	23	11	12	
≥ 60	17	9	8	
Gender				0.288
Male	29	13	16	
Female	11	7	4	
pT stage^a^				0.038
pT1 + pT2	13	3	9	
pT3 + pT4	27	17	11	
Tumor size (cm)				0.337
< 5	23	10	13	
≥ 5	17	10	7	
Location				0.749
Cardiac	17	9	8	
Non-cardiac	23	11	12	
pTNM stage^b^				0.025
I + II	17	5	12	
III + IV	23	15	8	
Differentiation				0.230
Poorly	37	20	17	
Well	3	0	3	

^a^*pTstage* pathological tumor stage, ^b^*pTNM* *stage* pathological tumor node metastasis stage

### Correlation and enrichment analyses of PGM1

**Fig. 2:**
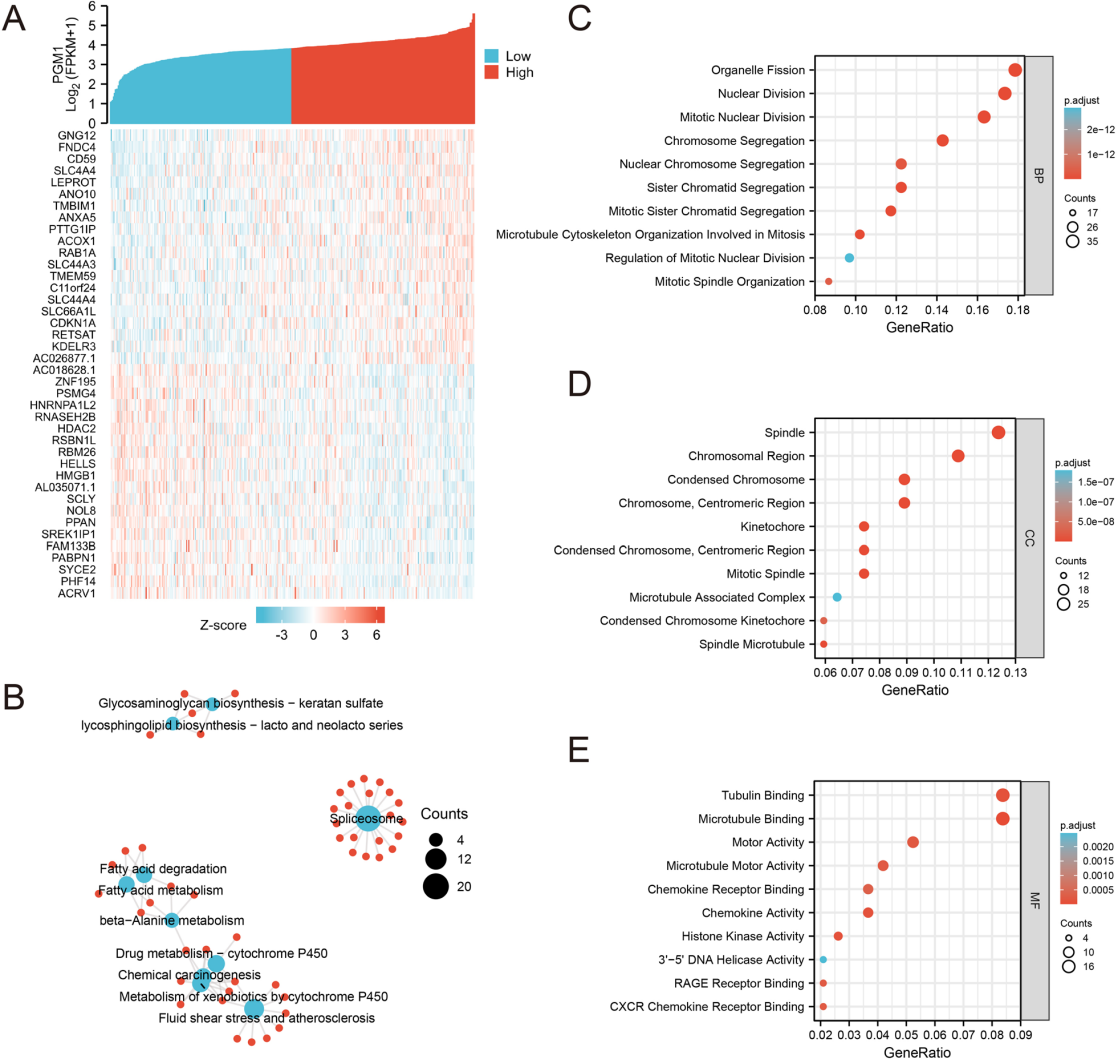
Correlation and enrichment analyses of PGM1. **a** The relative expression of PGM1 in GC tissues from TCGA database were shown above, which were divided into low and high groups according to the median expression. Top 20 genes that most positively associated with PGM1 expression and top 20 genes that most negatively associated with it were displayed below. **b** KEGG network to display top 10 pathways associated with PGM1 expression. **c** Top 10 BP terms that were significantly enriched in GO analysis. **d** Top 10 CC terms that were significantly enriched in GO analysis. **e** Top 10 MF terms that were significantly enriched in GO analysis. PGM1: Phosphoglucomutase 1; GC: gastric cancer; TCGA: The Cancer Genome Atlas; BP: biological process; CC: cellular component; MF: molecular function; KEGG: Kyoto Encyclopedia of Genes and Genomes; GO: Gene ontology

To further investigate the functions of PGM1 in GC progression, we employed TCGA database and screened 600 genes that were most positively or negatively associated with PGM1 expression, respectively. The top 40 of them were displayed by a heatmap (Fig. 2a). These 600 genes were enriched and analyzed by GO and KEGG analyses. GO analysis was divided into three terms, including biological process (BP), cellular component (CC) and molecular function (MF). The top 10 enriched pathways in each term were listed. The results suggested that PGM1 mainly regulated viability-related or proliferation-related signaling pathways. Most of them were concerned with mitosis and substance preparation for cell viability and proliferation (Fig. 2c–e). KEGG network indicated that PGM1 expression was closely correlated with glycometabolism and lipid metabolism. Otherwise, pathways of spliceosome and drug metabolism were also enriched (Fig. 2b). Collectively, PGM1 expression was associated with cell viability, proliferation and metabolism of GC.

### Knockdown of PGM1 attenuates the GC viability and proliferation under glucose deprivation

**Fig. 3:**
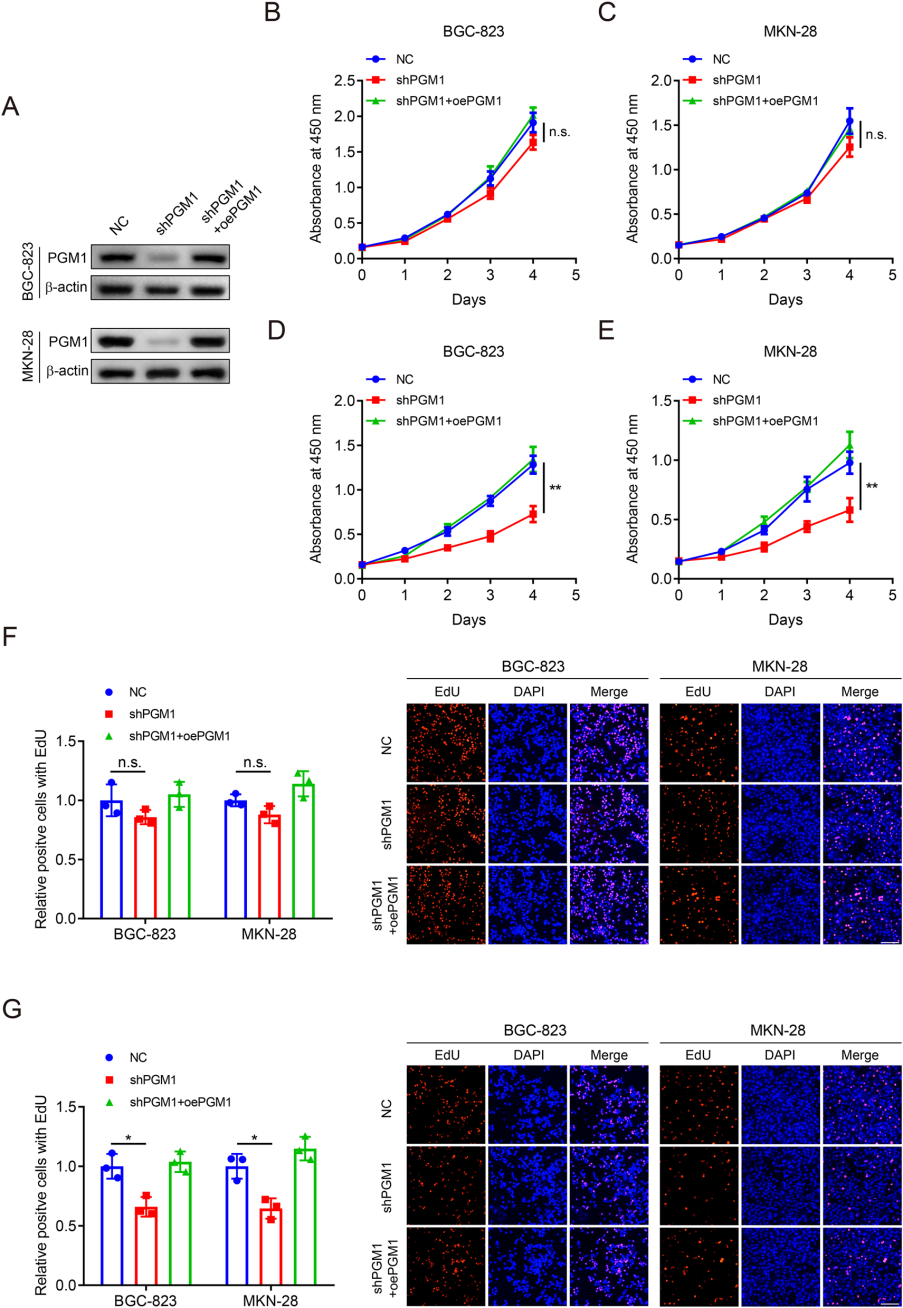
Knockdown of PGM1 attenuates GC viability and proliferation under glucose deprivation. **a** WB analysis to show the PGM1 expression in BGC-823 and MKN-28 cells stably carrying PGM1 shRNA or scrambled shRNA and additional transfection of PGM1 overexpression plasmids or vectors, respectively. **b**, **c** CCK-8 assay to measure viability of BGC-823 (**b**) and MKN-28 cells (**c**) as in (**a**) under the NG condition. **d**, **e** CCK-8 assay to measure viability of BGC-823 (**d**) and MKN-28 cells (**e**) as in (**a**) under the LG condition. **f** EdU assay to measure proliferation of cells as in (**b**, **c**). **g** EdU assay to measure proliferation of cells as in (**d**, **e**). Histograms are on the right. Scale bar: 100 μm. Data are shown as the mean ± SD. *P < 0.05, **P < 0.01, n.s. no significant. PGM1: Phosphoglucomutase 1; NG: normal glucose, 10 mM glucose in medium; LG: low glucose, 2.5 mM glucose in medium; NC: negative control; WB: western blot; CCK-8: cell counting kit-8; EdU: 5-ethynyl-2-deoxyuridine; DAPI: 4′,6-diamidino-2-phenylindole; SD: standard deviation

Since the close association between PGM1 expression and GC development, we next explored the oncological functions. BGC-823 and MKN-28 cells were selected for further experiments due to their high expression of PGM1. A shRNA targeting PGM1 and the corresponding overexpression plasmids were synthesized. BGC-823 and MKN-28 cells were infected with lentivirus carrying the shRNA targeting PGM1. The regulatory effects of the shRNA and additional transfection of overexpression plasmids on PGM1 expression were examined by WB analysis (Fig. 3a). CCK-8 assay demonstrated that PGM1 knockdown only slightly decreased cell viability under the NG condition in BGC-823 and MKN-28 cells without statistical difference. However, when cells were cultivated in LG medium, PGM1 expression downregulation significantly attenuated cell viability. To assess the off-target effects of the shRNA, we restored PGM1 expression by additional transfection of PGM1 overexpression plasmids, which rescued the viability of GC cells (Fig. 3b–e). The results suggested that the shRNA targeting PGM1 had no off-target effects on viability. The PGM1-mediated regulation of proliferation was confirmed by the EdU assay and the data exhibited similar results with cell viability (Fig. 3f, g). Our findings suggested that PGM1 expression downregulation could suppress GC viability and proliferation under glucose deprivation in vitro.

### Altered PGM1 expression affects glucose and lipid metabolism under glucose deprivation

**Fig.4:**
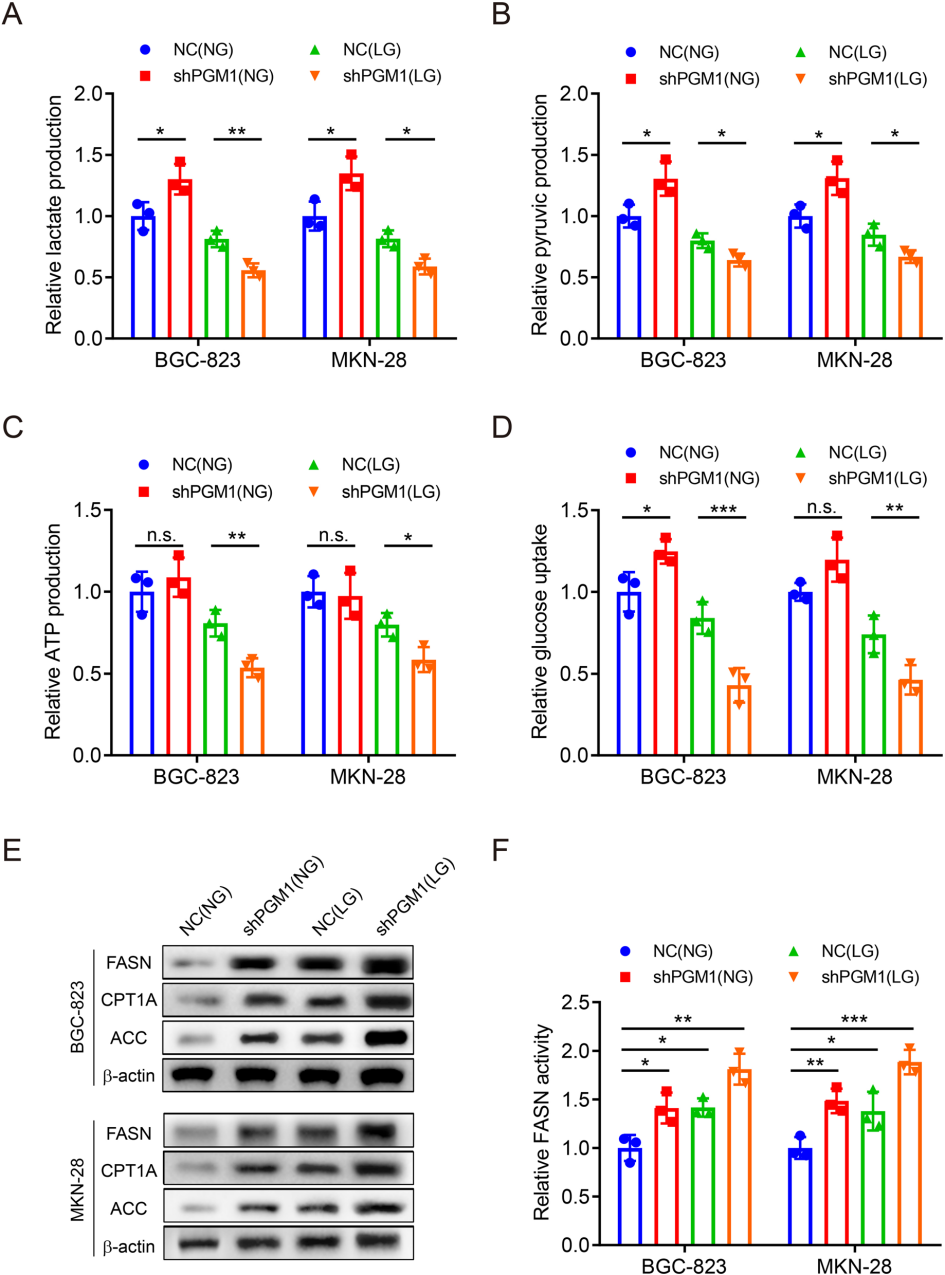
Altered PGM1 expression affects glucose and lipid metabolism under glucose deprivation. **a**–**d** Colorimetry assays to measure glycolysis of BGC-823 and MKN-28 cells with knockdown of PGM1 or NC under the NG or LG condition, including lactate acid (**a**), pyruvic acid (**b**), ATP (**c**) production and glucose uptake capability (**d**). **e** WB assay to show protein expression levels of cells as in (**a**–**d**). **f** Colorimetry assay to measure FASN activity of cells as in (**a**–**d**). Data are shown as the mean ± SD. *P < 0.05, **P < 0.01, ***P < 0.001, n.s. no significant. PGM1: Phosphoglucomutase 1; FASN: Fatty Acid Synthase; CPT1A: Carnitine Palmitoyltransferase 1A; ACC: Acetyl CoA Carboxylase; NC: negative control; NG: normal glucose, 10 mM glucose in medium; LG: low glucose, 2.5 mM glucose in medium; SD: standard deviation

Since the close relationship between PGM1 and glucose metabolism, we aimed to explore the alterations in glycolysis levels after PGM1 knockdown. Lactate, pyruvic acid, and ATP are important metabolites. Glucose uptake is the first step of glucose metabolism. We found that PGM1 knockdown in NG medium led to mild increases in lactate, pyruvic acid production and glucose uptake, but there was no significant change in ATP production. These results showed that suppression of PGM1 under the glucose-sufficient condition could not effectively affect the energy supply in GC cells. However, sharp decreases in the lactate, ATP, pyruvic acid production and glucose uptake were observed after knockdown of PGM1 under the LG condition (Fig. 4a–d). The data proved that PGM1 acted as a critical role in maintaining glycolysis under the LG condition.

Next, we investigated lipid metabolism regulated by PGM1 and glucose supply. WB analysis was used to measure the expression of FASN, Carnitine Palmitoyltransferase 1A (CPT1A) and Acetyl CoA Carboxylase (ACC) in lipid metabolism. FASN, CPT1A and ACC were upregulated by PGM1 knockdown or glucose deprivation, respectively. Combination of these two factors significantly enhanced their expression compared to the groups with single intervention (Fig. 4e). FASN activity in BGC-823 and MKN-28 was also mostly enhanced by PGM1 knockdown under the LG condition (Fig. 4f). Collectively, the results suggested that PGM1 could regulate the balance between glycolysis and lipid metabolism. PGM1 expression downregulation suppressed glycolysis levels and facilitated lipid metabolism in GC cells under the LG condition.

### Orlistat inhibits lipid metabolism but promotes glycolysis in GC

**Fig.5:**
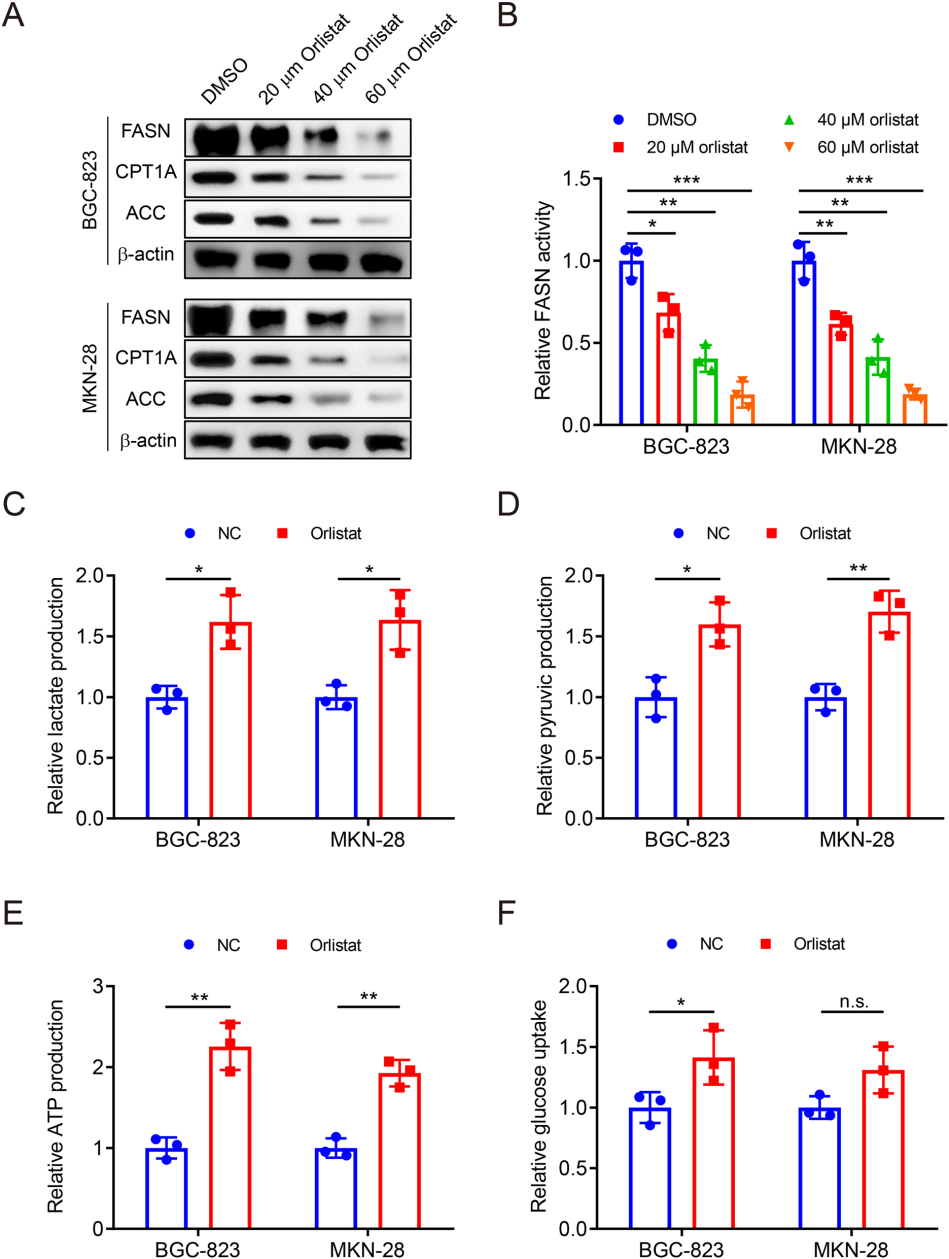
Orlistat inhibits lipid metabolism but promotes glycolysis in GC. **a** WB assay to show protein expression levels of BGC-823 and MKN-28 cells after treatment of DMSO, 20 μM, 40 μM or 60 μM orlistat for 24 h under the NG condition. **b** Colorimetry assay to measure FASN activity of cells as in (**a**). **c**–**f** Colorimetry assays to measure glycolysis in GC cells after treatment of DMSO or 60 μM orlistat for 24 h under the NG condition, including lactate acid (**c**), pyruvic acid (**d**), ATP (**e**) production and glucose uptake capability (**f**). Data are shown as the mean ± SE. *P < 0.05, **P < 0.01, ***P < 0.001, n.s. no significant. PGM1: Phosphoglucomutase 1; FASN: Fatty Acid Synthase; CPT1A: Carnitine Palmitoyltransferase 1A; ACC: Acetyl CoA Carboxylase; NG: normal glucose, 10 mM glucose in medium; NC: negative control; DMSO: dimethyl sulphoxide; SD: standard deviation

Orlistat was initially designed to suppress lipid metabolism for weight loss. To confirm the role of orlistat in GC therapy, we treated BGC-823 and MKN-28 cells with 0, 20, 40, 60 μM orlistat. FASN, CPT1A and ACC expression levels were reduced (Fig. 5a) and FASN activity was also inhibited with increasing concentration of orlistat (Fig. 5b). Surprisingly, we found that orlistat reversely increased lactate, ATP, pyruvic acid production and glucose uptake (Fig. 5c–f). These results revealed that orlistat treatment inhibited lipid metabolism, whereas promoted glycolysis levels in GC cells.

### PGM1 expression downregulation enhances orlistat-induced apoptosis under glucose deprivation

**Fig.6:**
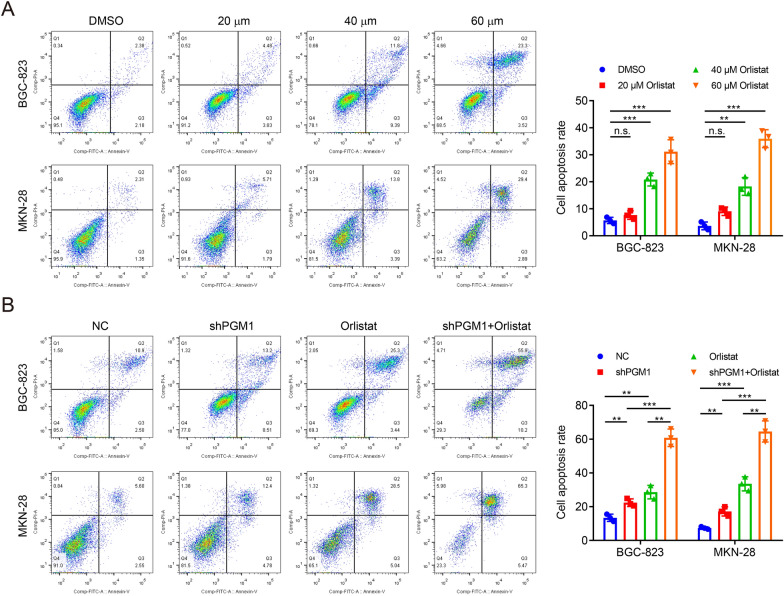
PGM1 expression downregulation enhances orlistat-induced apoptosis under glucose deprivation. **a** Flow cytometry to detect the apoptosis of BGC-823 and MKN-28 cells treated with DMSO, 20 μM, 40 μM or 60 μM orlistat for 24 h under the NG condition. **b** Flow cytometry to detect the apoptosis of BGC-823 and MKN-28 cells with knockdown of PGM1 or NC and treated with DMSO or 60 μM orlistat for 24 h under the LG condition. Data are shown as the mean ± SD. **P < 0.01, ***P < 0.001, n.s. no significant. PGM1: Phosphoglucomutase 1; NC: negative control; DMSO: dimethyl sulphoxide; NG: normal glucose, 10 mM glucose in medium; LG: low glucose, 2.5 mM glucose in medium; SD: standard deviation

We next investigated anti-cancer effects of orlistat in GC. The flow cytometry assay demonstrated that orlistat treatment led to apoptosis of BGC-823 and MKN-28 cells and that there was a significant dose–effect relationship (Fig. 6a). Since the complementary roles of orlistat and PGM1 knockdown in metabolic regulation, it was speculated that PGM1 expression downregulation could reverse the glycolysis enhancement caused by orlistat under the LG condition, which might lead to the sensitization to orlistat in GC cells. Data showed that under the LG condition, PGM1 knockdown induced cell apoptosis. The anticancer effects of orlistat were significantly augmented after PGM1 knockdown compared to the group of single treatment (Fig. 6b). Our findings suggested that PGM1 expression downregulation enhanced the effects of orlistat, possibly through attenuating both glucose and lipid metabolisms, impairing the energy compensation caused by the single factor.

### The combination of orlistat and PGM1 expression downregulation suppresses tumor growth and metastasis in vivo

**Fig.7:**
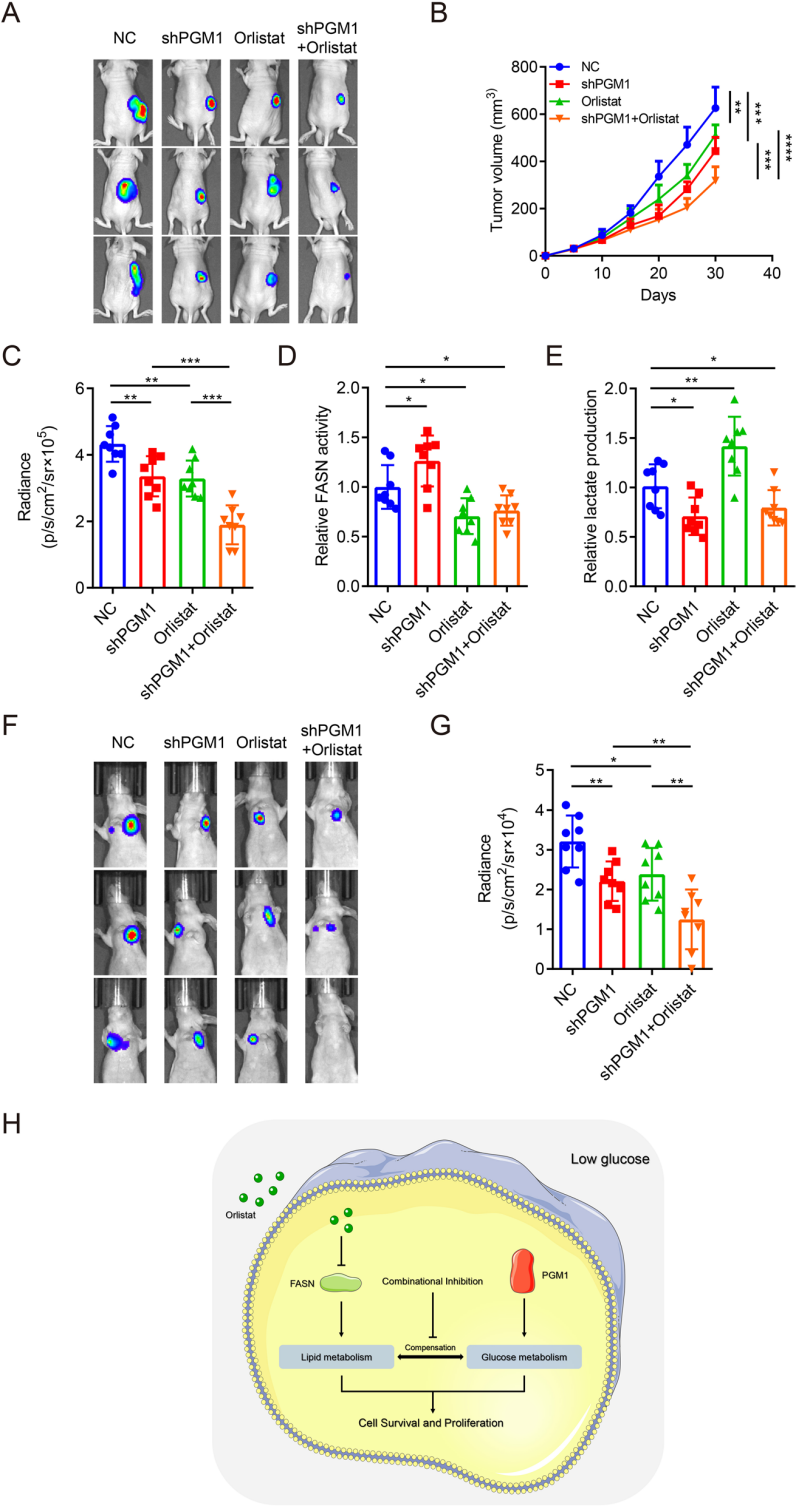
The combination of orlistat and PGM1 expression downregulation suppresses tumor growth and metastasis in vivo. **a** Representative bioluminescence images at 30 days after subcutaneous injection of luc-BGC-823 cells with knockdown of PGM1 or NC and treated with 240 μg/g of orlistat or the corresponding solvent daily. 3 mice in each group were displayed. **b** Curves of tumor volumes as in (**a**) at the indicated time. **c** Luminescence signals in (**a**) represented by overlaid false-color images with the signal intensity. **d** Lactate production was determined in the tumor tissues from (**a**). **e** FASN activity was determined in the tumor tissues from (**a**). **f** Representative bioluminescence images at 30 days after tail vein injection of luc-BGC-823 cells with knockdown of PGM1 or NC and treated with 240 μg/g of orlistat or the corresponding solvent daily. 3 mice in each group were displayed. **g** Luminescence signals in (**f**) represented by overlaid false-color images with the signal intensity. **h** Schematic illustration of mechanisms of PGM1 knockdown plus orlistat treatment in GC therapy. Data are shown as the mean ± SD. *P < 0.05, **P < 0.01, ***P < 0.001. GC: gastric cancer; PGM1: Phosphoglucomutase 1; FASN: Fatty Acid Synthase; NC: negative control; SD: standard deviation

To promote the clinical translation of this regimen, we generated nude mice with subcutaneous tumors and lung metastasis. The volumes of the tumors derived from luc-BGC-823 cells with stable PGM1 knockdown were smaller than those derived from the control group. Treatment with orlistat could also decrease the tumor burden. Combination of the two approaches achieved better efficacies than either approach alone (Fig. 7a–c). For the metabolic alterations, PGM1 expression downregulation suppressed lactate production and facilitated FASN activity in tumor tissues. Orlistat treatment showed the opposite effects. Combination use led to simultaneous attenuation of lactate production and FASN activity (Fig. 7d, e). Similar with the results of the model of subcutaneous tumors, the metastatic capability was also suppressed by PGM1 knockdown and orlistat treatment. The effects of orlistat on metastasis suppression were enhanced after PGM1 knockdown (Fig. 7f, g). In summary, our findings revealed a novel therapeutic regimen for GC. Orlistat could effectively induce cancer cell apoptosis and PGM1 knockdown under glucose deprivation enhanced this anticancer effect in vitro and in vivo by inhibiting the glucose and lipid metabolism (Fig. 7h).

## Discussion

Cancer cells universally undergo reprogramming of glucose and lipid metabolisms, which can provide energy supply to support the proliferative state and resist against harsh environments. These two metabolic pathways are both activated and regulated by many oncogenes in GC [13–15]. Inhibiting critical targets of cell metabolism effectively suppresses malignant behaviors and becomes a novel strategy of cancer treatment [16, 17]. Some small-molecule inhibitors that were originally developed for treatment of metabolic diseases have exhibited potentials in cancer inhibition [11, 18]. However, many studies focused on only one type of metabolism. Inhibiting one metabolic pathway might not be fully efficacious in clinical practice due to the compensation of other metabolic pathways. Importantly, glucose deprivation is one of the features of the microenvironment where tumors grow in vivo. An alteration in glucose concentration can drive cancer cells to regulate intracellular signaling pathways and rely on other energy sources [19, 20]. The evidence proved that cancer cells actively undergo metabolic reprogramming to adapt to nutrient starvation and environmental stress.

PGM1 reversibly regulates the catabolism and anabolism of glycogen under physiological conditions [21]. Deficiency of PGM1 expression during embryonic development can lead to inherited metabolic disorders [22]. PGM1 was also reported to promote progression in lung and ovarian cancer [9, 10]. We notice that PGM1 inhibited malignant progression of hepatocellular carcinoma [23], which seems to contradict conclusions of other studies. The inconsistence may result from differences in cancer types or cultivation conditions. The role of PGM1 in GC has not been clearly identified. In this study, qRT-PCR, IHC and WB analyses showed that PGM1 expression was upregulated in GC tissues and cell lines. The positive correlations between PGM1 expression and pT stage, pTNM stage were also observed in the included patients. The data from the Kaplan–Meier Plotter database showed that higher PGM1 expression was correlated with poor prognosis of patients with GC at stage III, poorly differentiated GC, or the intestinal type of it [24]. PGM1 had great potentials as a diagnostic biomarker and prognostic factor.

The enrichment analysis was conducted to investigate the functions and signaling pathways of PGM1. PGM1 was associated with cell viability, proliferation and metabolism. It suggested that PGM1 might regulate GC growth through metabolic reprogramming. The experiments both in vitro and in vivo were performed for further investigation. Consistent with previous studies [9, 10], downregulation of its expression significantly inhibited cancer progression under glucose deprivation. For metabolic alterations, PGM1 knockdown led to suppression of glycolysis, while lipid metabolism was moderately enhanced. This metabolic conversion might be explained by the compensatory effects of lipid metabolism under nutrient-insufficient conditions.

Orlistat is a classical inhibitor of FASN and has exhibited its anti-cancer effects in the past decade. Chuang et al. revealed that orlistat sensitized prostate cancer to radiotherapy via FASN/NF-κB pathway [25]. Almeida et al. constructed mouse models of spontaneous melanoma metastasis. They reported that orlistat attenuated metastatic capability of melanoma cell lines and activated immune response [26]. Our findings proved that orlistat treatment reduced lipid metabolism in GC cells. There were significant dose-dependent effects on orlistat-induced apoptosis. Interestingly, glycolytic levels were increased after orlistat treatment. Accumulating evidence indicated that glycolysis enhanced drug resistance and cancer survival [27, 28], which may be one of the factors that impairs orlistat effectiveness. Given that the complementary functions of PGM1 and orlistat, we speculated that PGM1 knockdown could enhance orlistat efficacy by combinational suppression of glucose and lipid metabolisms. The results showed that PGM1 knockdown under the LG condition enhanced apoptosis induced by orlistat in GC cells. Similarly, in vivo experiments indicated that PGM1 knockdown or orlistat treatment decreased GC growth and metastasis. The combinational regimen outperformed the single approach through blocking glucose and lipid metabolisms in GC.

However, there are some limitations in this study. We solely focused on the relationships between metabolism and cancer progression. The mechanisms by which this combinational regimen inhibited GC and metabolic pathways are still unclear. Accumulating studies reported critical regulators in metabolic reprogramming, such as KRAS, c-Myc and NRF2 [29–32]. More experiments should be conducted to investigate the interconnection and regulatory relationships with PGM1. Identification of underlying mechanisms can promote better understanding of this novel regimen. Furthermore, the potential off-target effects of orlistat need further exploration. Clinical trials are also required to confirm the value of this regimen in GC treatment.

## Conclusions

Taken together, our findings identified the oncogenic role of PGM1 in GC for the first time, and validated that PGM1 has the potential for GC diagnosis and prognostic prediction. Combination of PGM1 knockdown and orlistat treatment was proposed based on the complementary mechanisms in metabolic reprogramming. Orlistat treatment combined with inhibition of PGM1 effectively suppressed GC progression both in vitro and in vivo. This strategy provides a novel insight for the development of anti-metabolic therapy.

## Supplementary Information

**Additional file 1:** Original data of WB and EdU pictures.

## Data Availability

The datasets generated and/or analyzed during the current study are available from the corresponding author on reasonable request.

## Abbreviations

GC: Gastric cancer
PGM1: Phosphoglucomutase 1
FASN: Fatty acid synthase
CPT1A: Carnitine Palmitoyltransferase 1A
ACC: Acetyl CoA Carboxylase
TCGA: The Cancer Genome Atlas
GO: Gene ontology
BP: Biological process
CC: Cellular component
MF: Molecular function
KEGG: Kyoto Encyclopedia of Genes and Genomes
CCK-8: Cell counting kit-8
EdU: 5-Ethynyl-2-deoxyuridine
qRT-PCR: Quantitative real-time PCR
WB: Western blot
OS: Overall survival
PFS: Progression-free survival
NC: Negative control
NG: Normal glucose, 10 mM glucose in medium
LG: Low glucose, 2.5 mM glucose in medium
DMSO: Dimethyl sulphoxide
pT stage: Pathological tumor stage
pTNM stage: Pathological tumor node metastasis stage
PBS: Phosphate buffer saline
PI: Propidium iodide
DAPI: 4′,6-Diamidino-2-phenylindole
SD: Standard deviation
