# Supplementary material for: Knockdown of PGM1 enhances anticancer effects of orlistat in gastric cancer under glucose deprivation
Source: Cancer Cell Int. 2021 Sep 10;21:481. doi: 10.1186/s12935-021-02193-3 (PMC8434706; doi:10.1186/s12935-021-02193-3)
Supplement: Supplementary file 1 — Additional file 1: Original data of WB and EdU pictures. [file 12935_2021_2193_MOESM1_ESM.docx]

**EdU pictures**

**Fig. 3f**

BGC-823-NC-DAPI


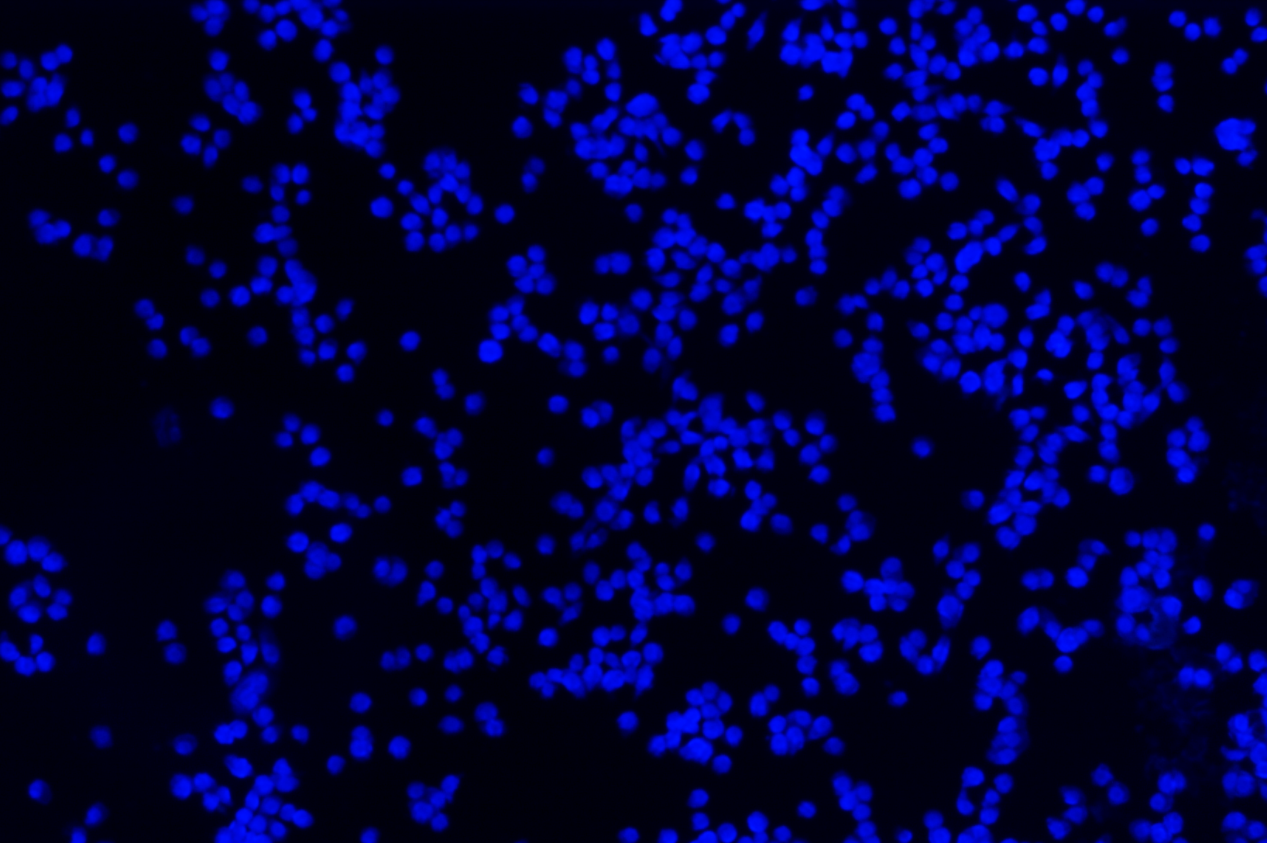


BGC-823-NC-EdU

**
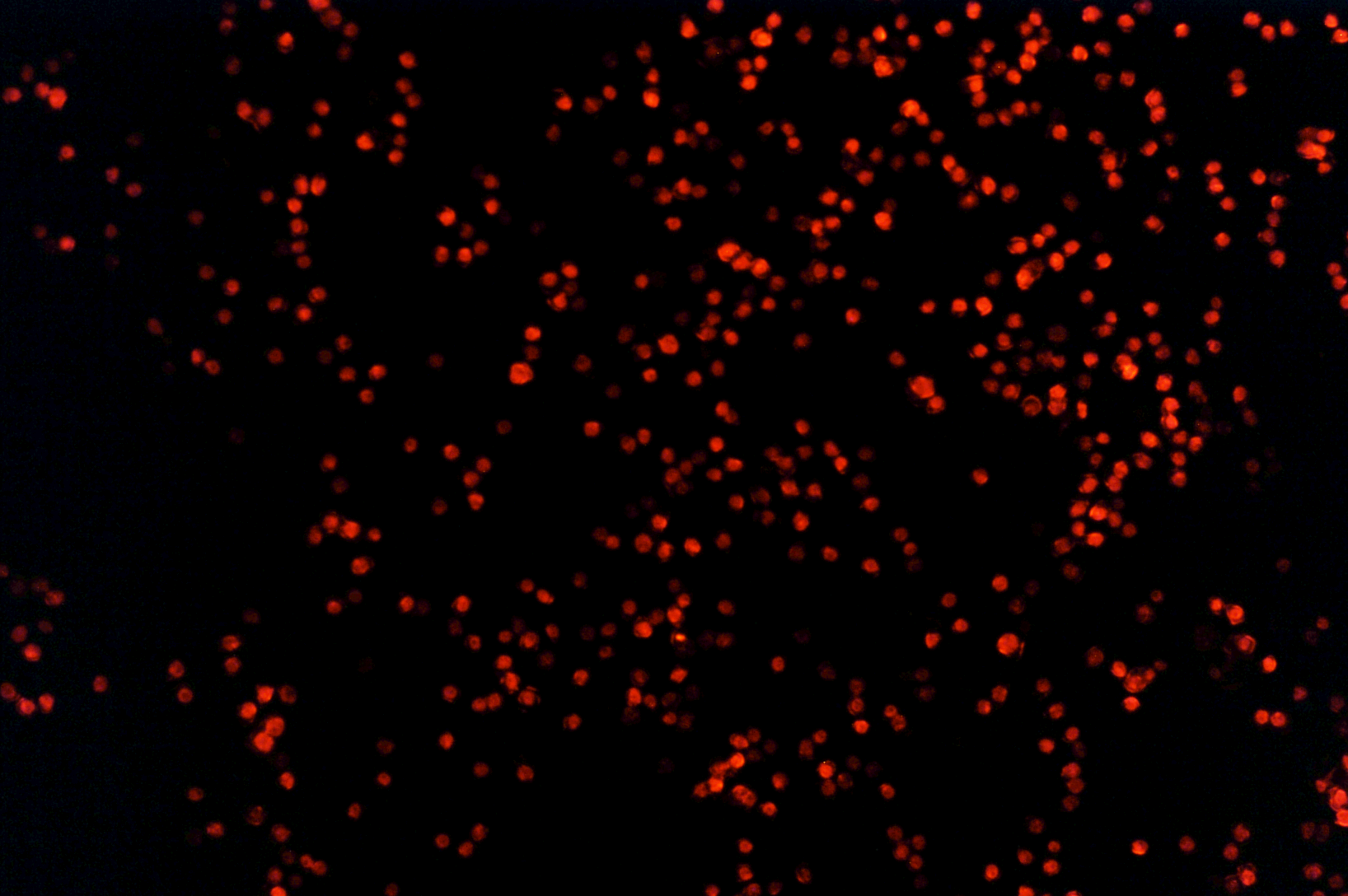
**

BGC-823- shPGM1-DAPI

**
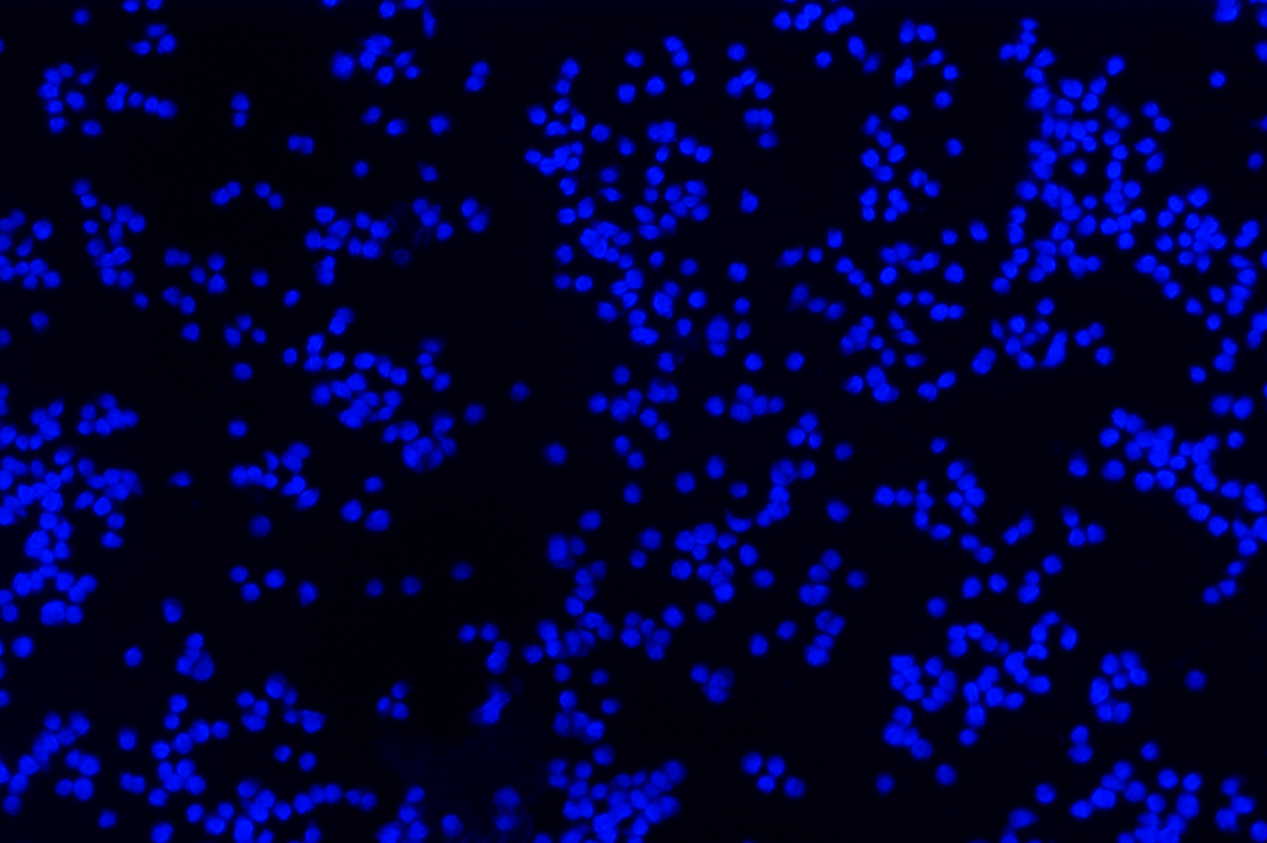
**

BGC-823-shPGM1-EdU

**
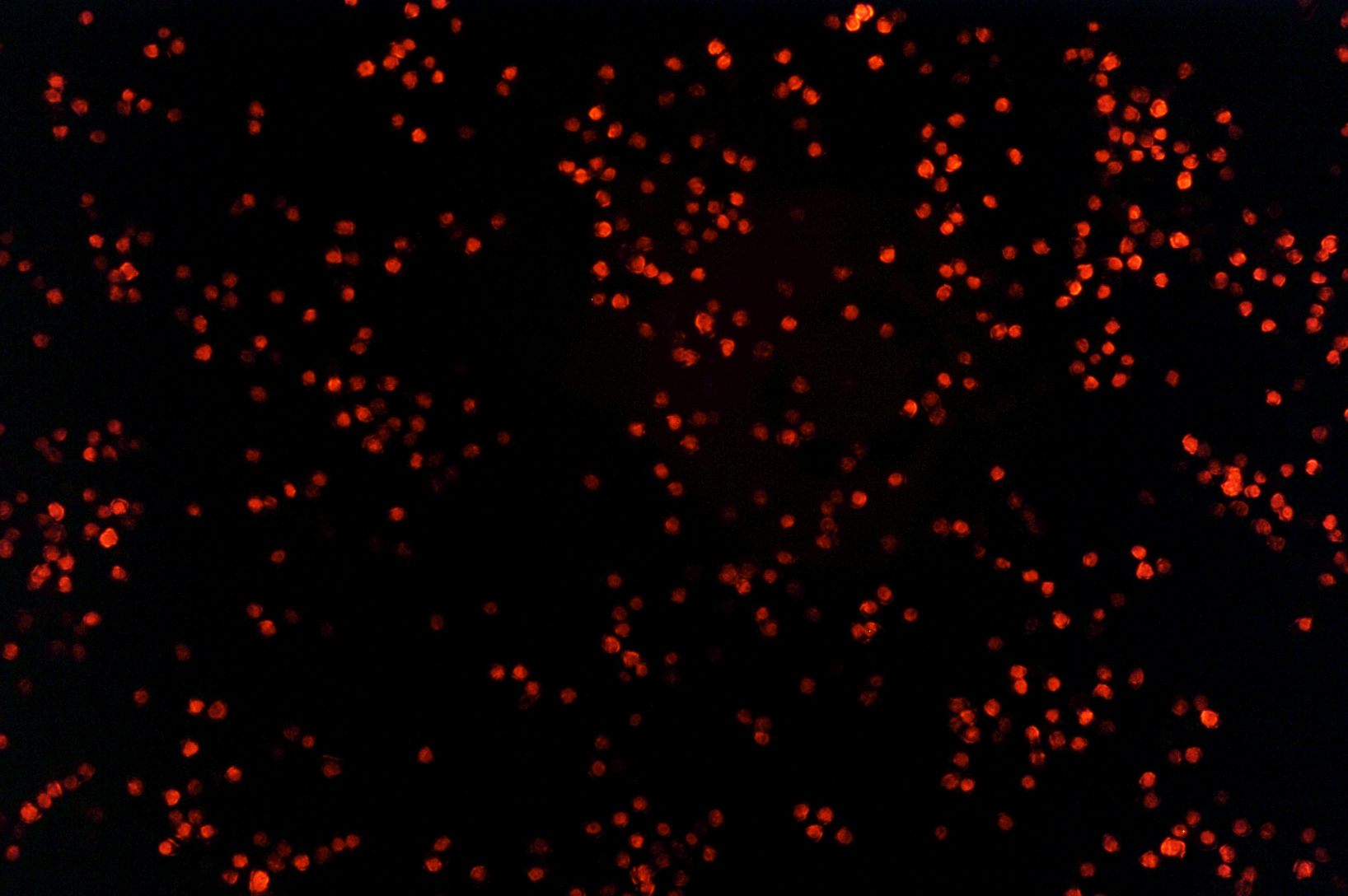
**

BGC-823- shPGM1+oePGM1-DAPI

**
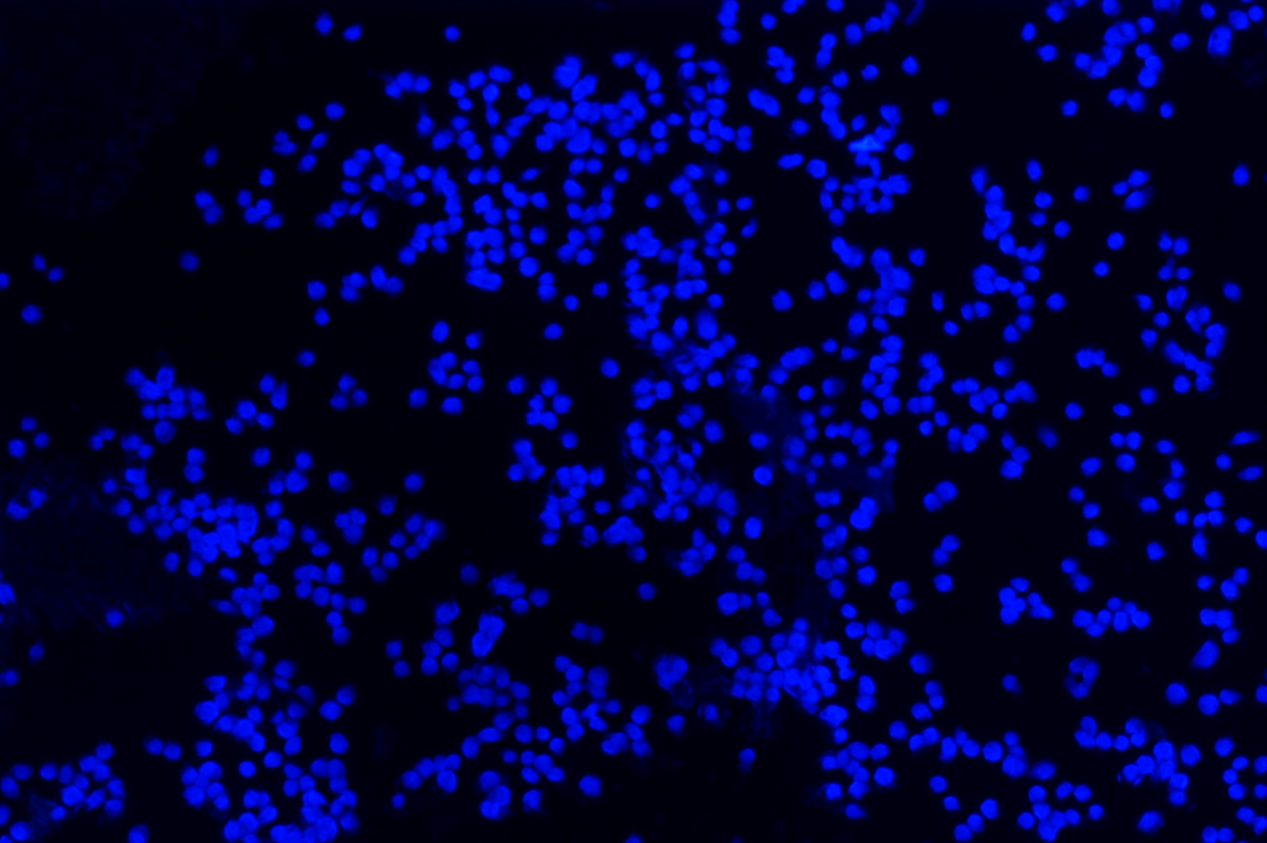
**

BGC-823- shPGM1+oePGM1-EdU

**
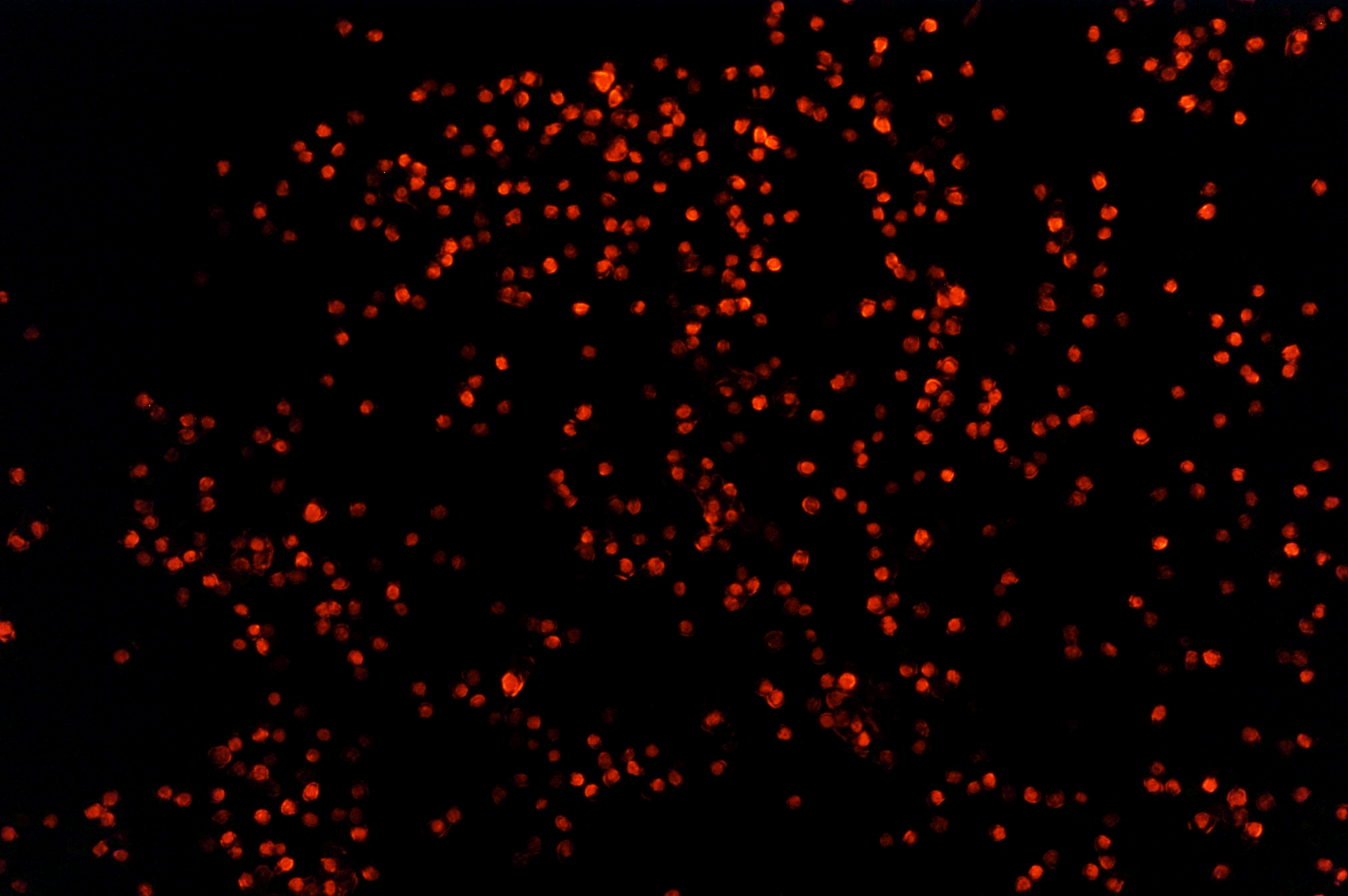
**

MKN-28-NC-DAPI


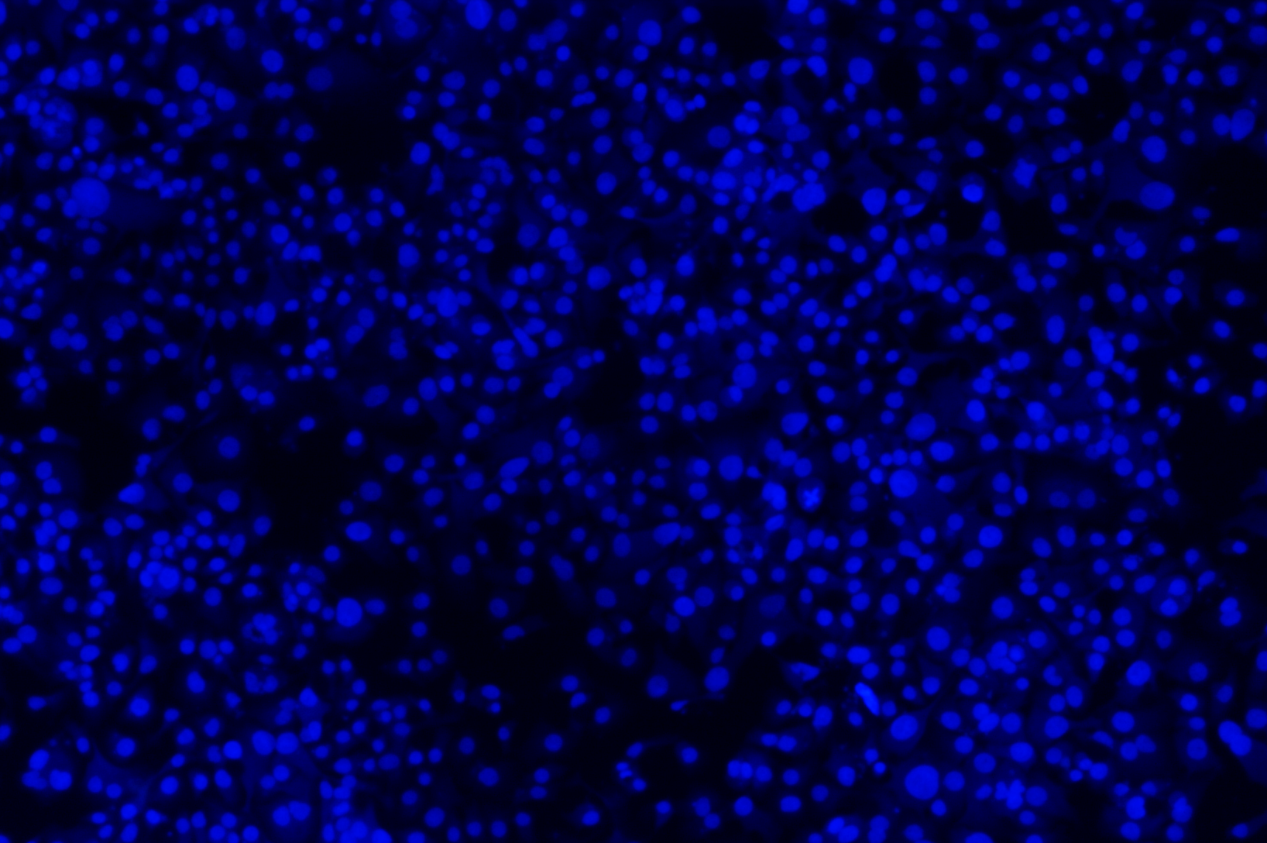


MKN-28-NC-EdU


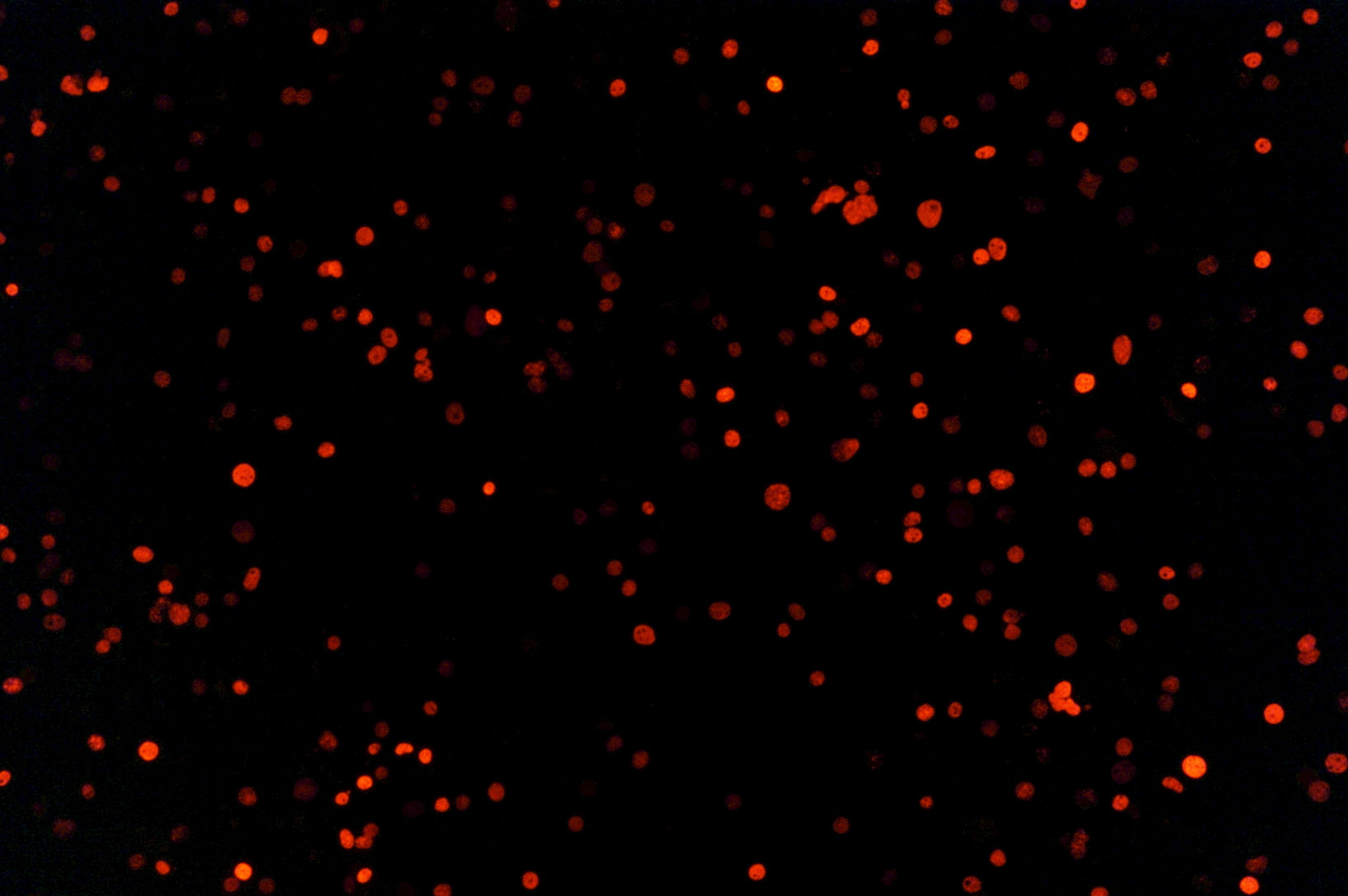


MKN-28- shPGM1-DAPI


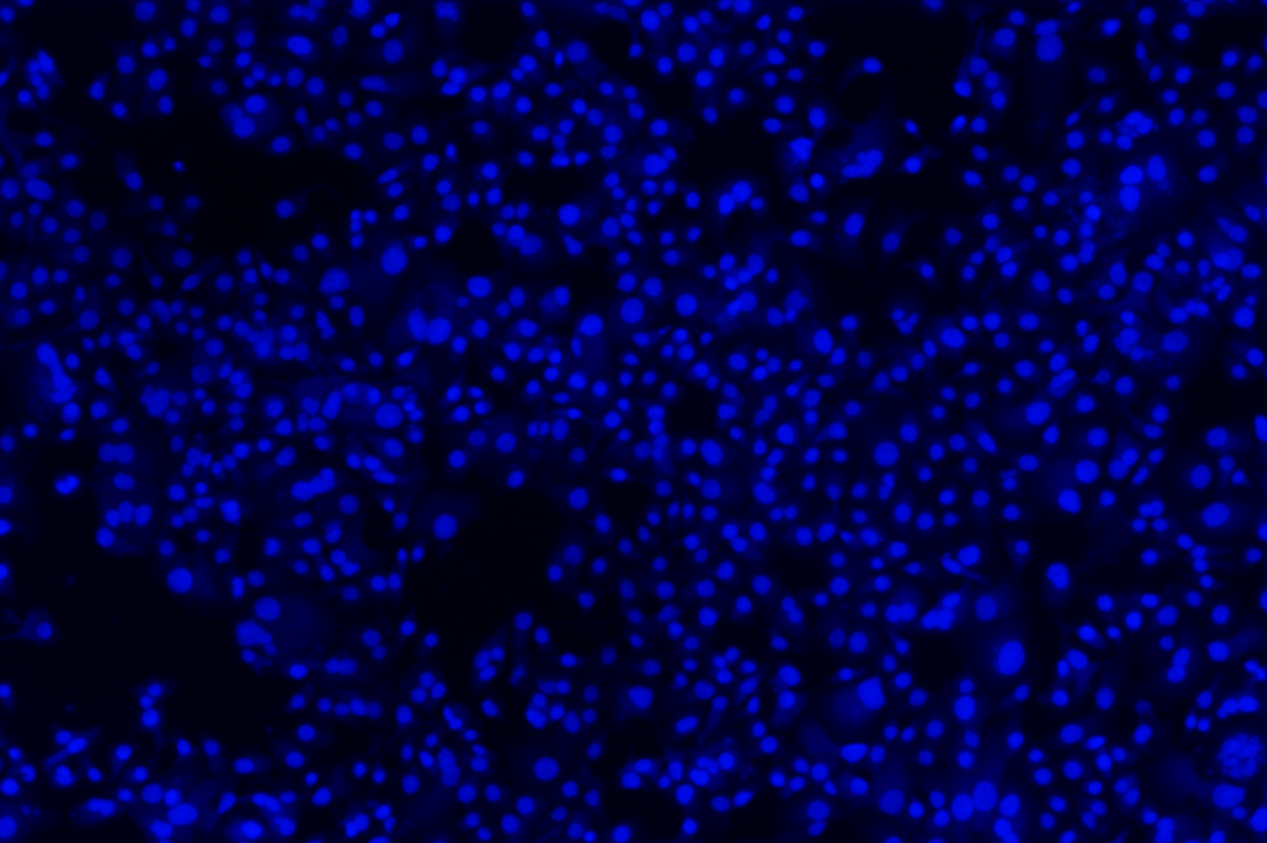


MKN-28-shPGM1-EdU


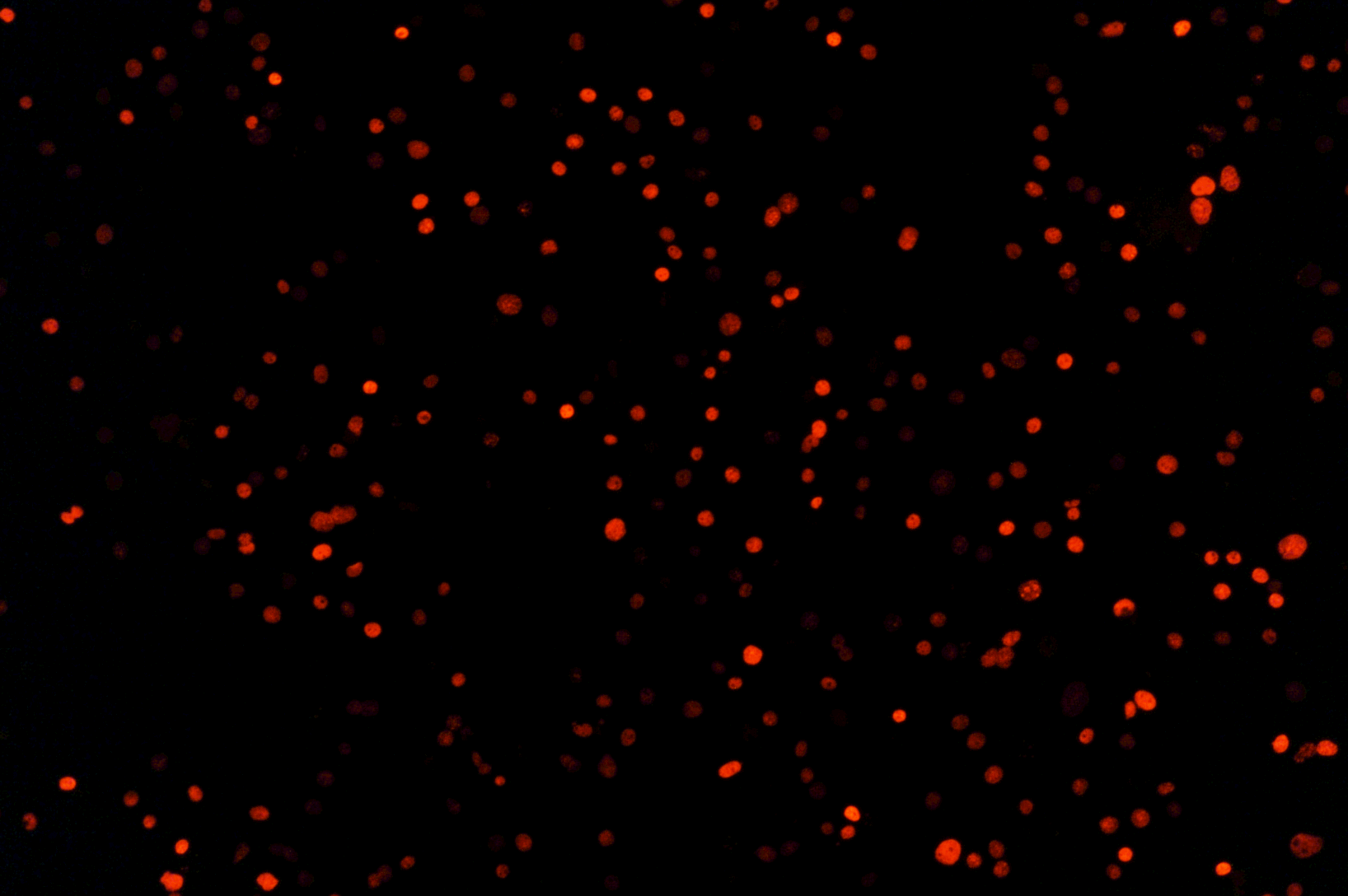


MKN-28- shPGM1+oePGM1-DAPI


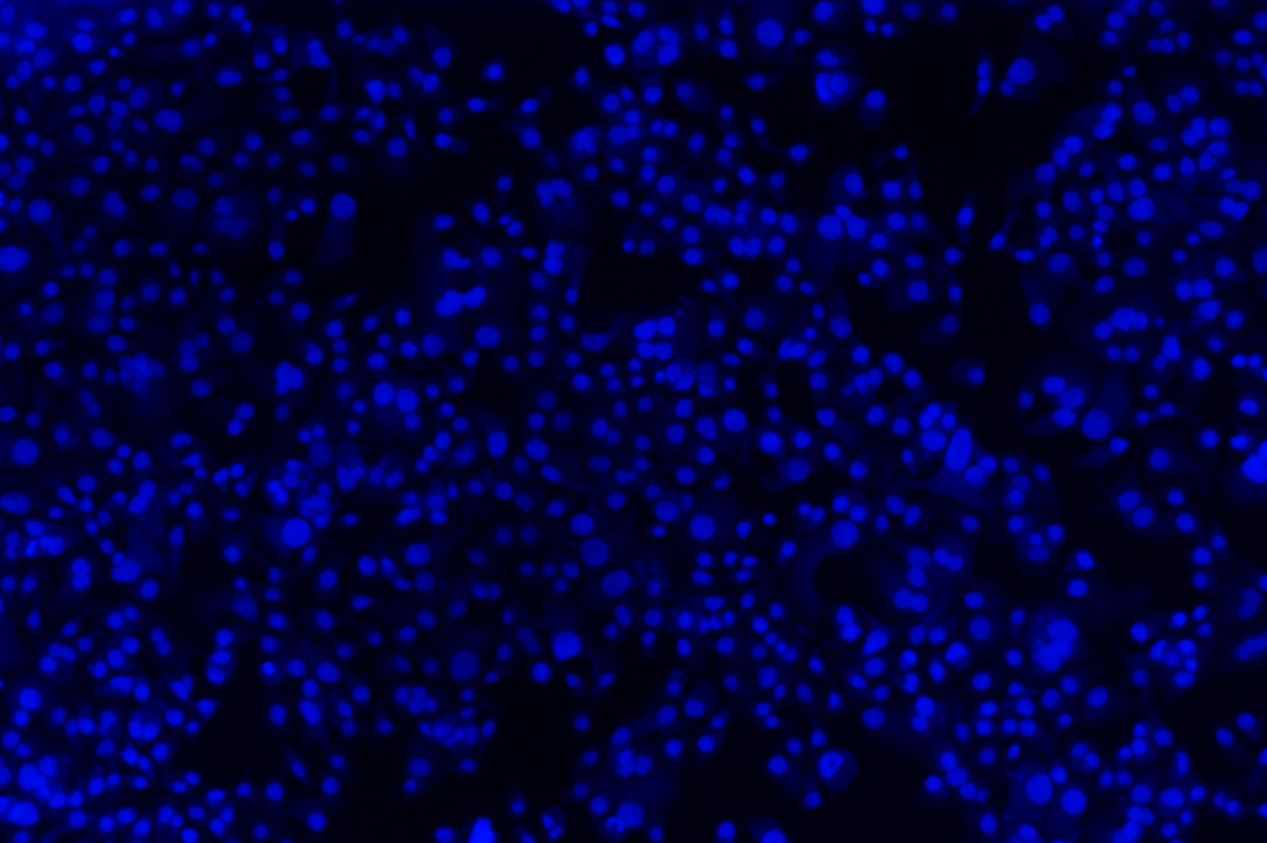


MKN-28- shPGM1+oePGM1-EdU


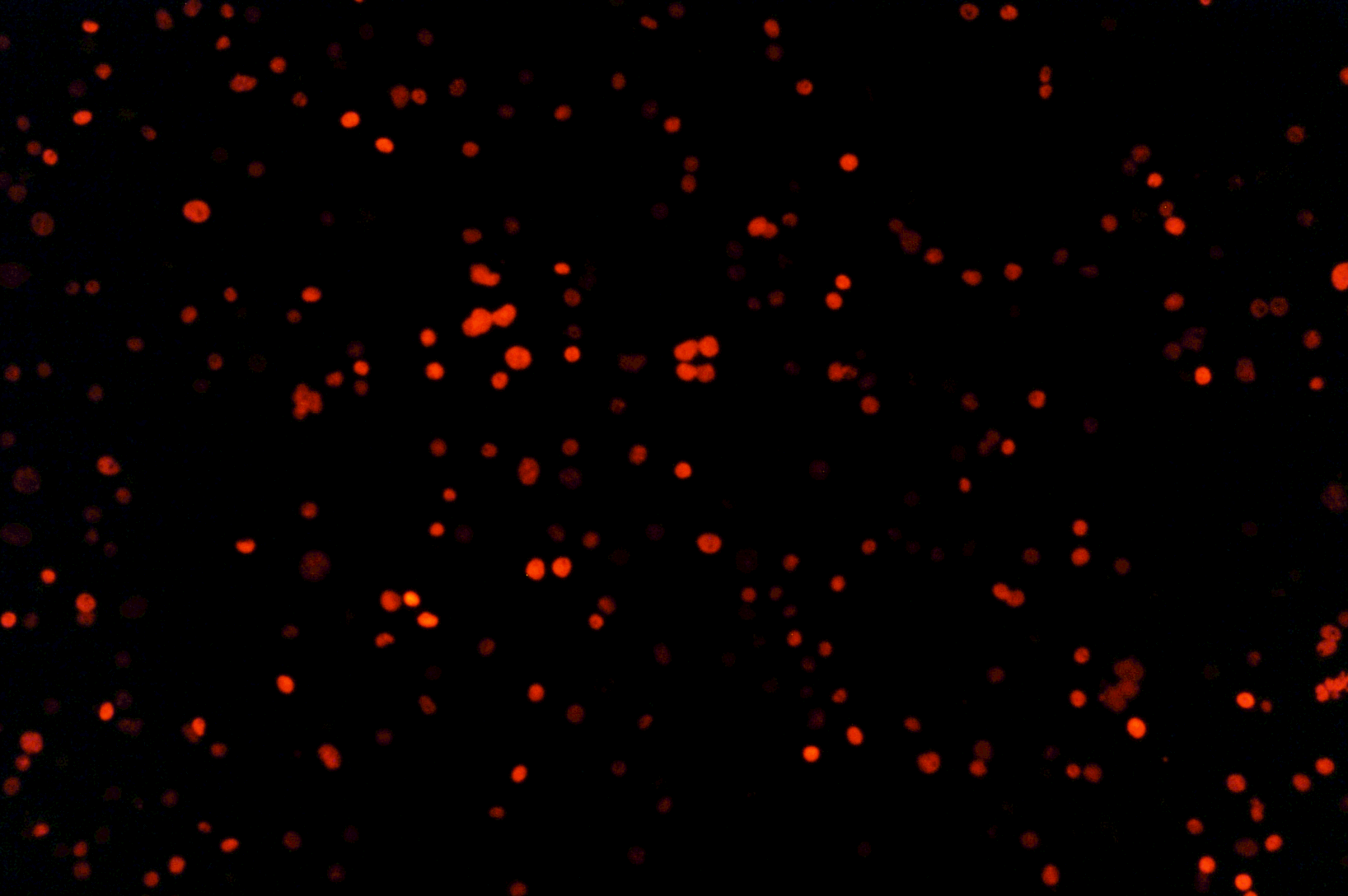


**Fig. 3g**

BGC-823-NC-DAPI

**
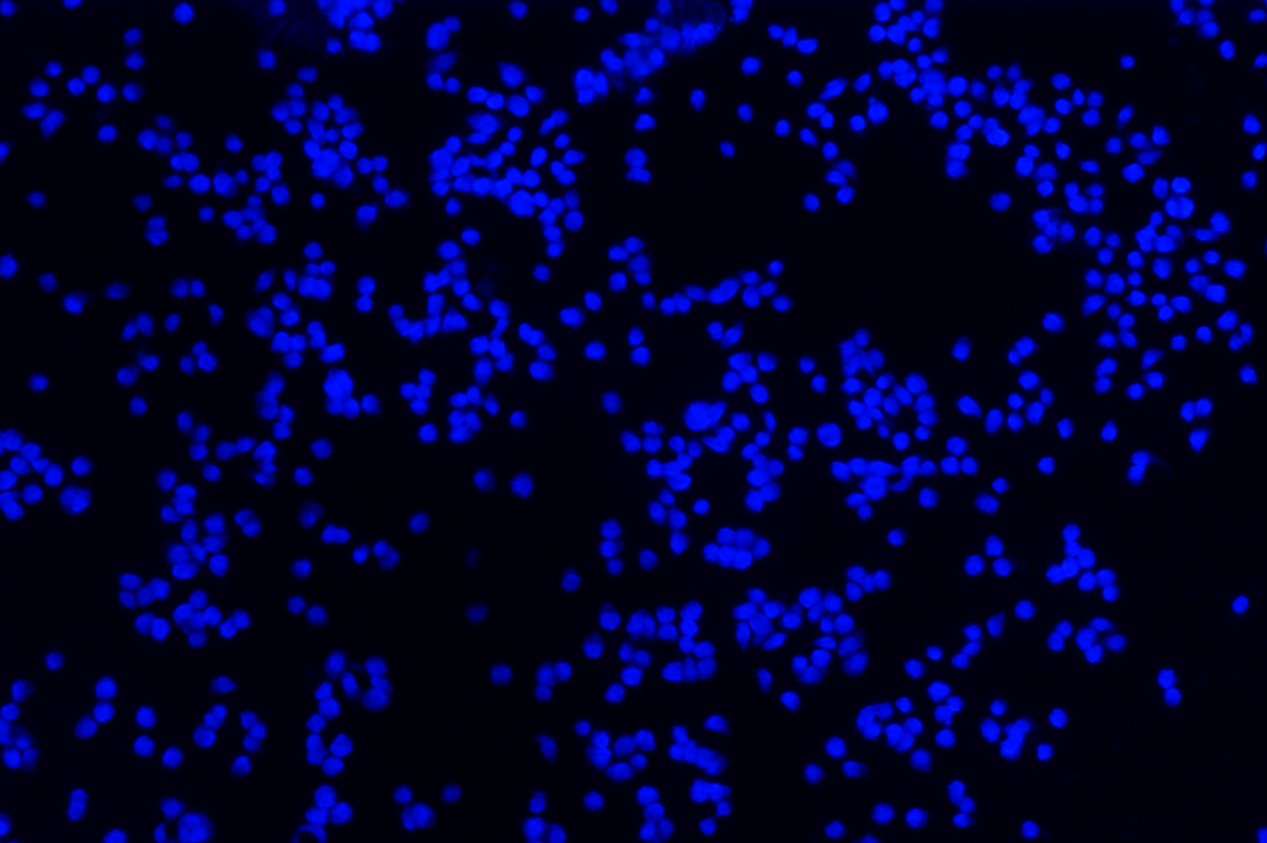
**

BGC-823-NC-EdU

**
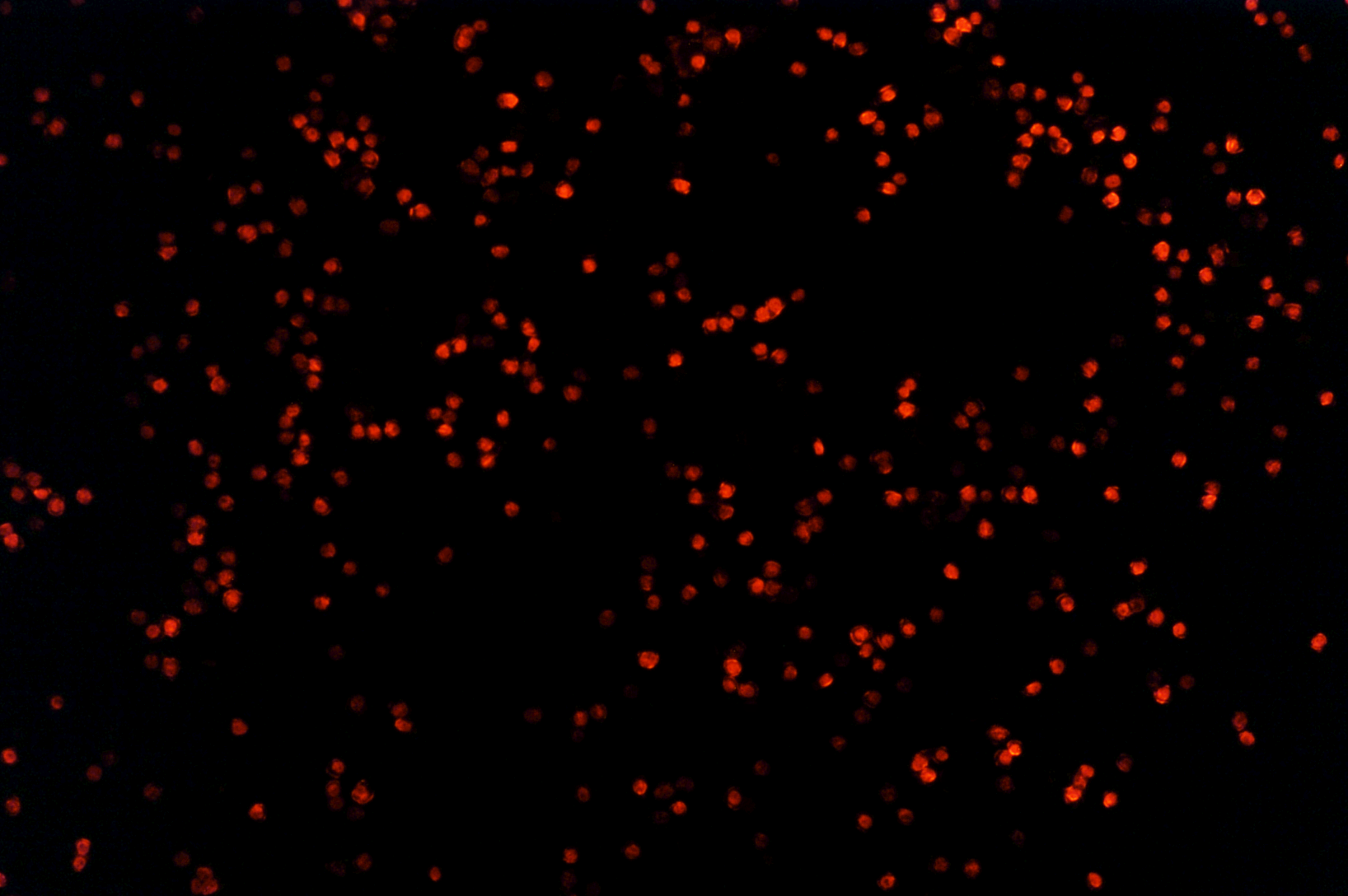
**

BGC-823- shPGM1-DAPI

**
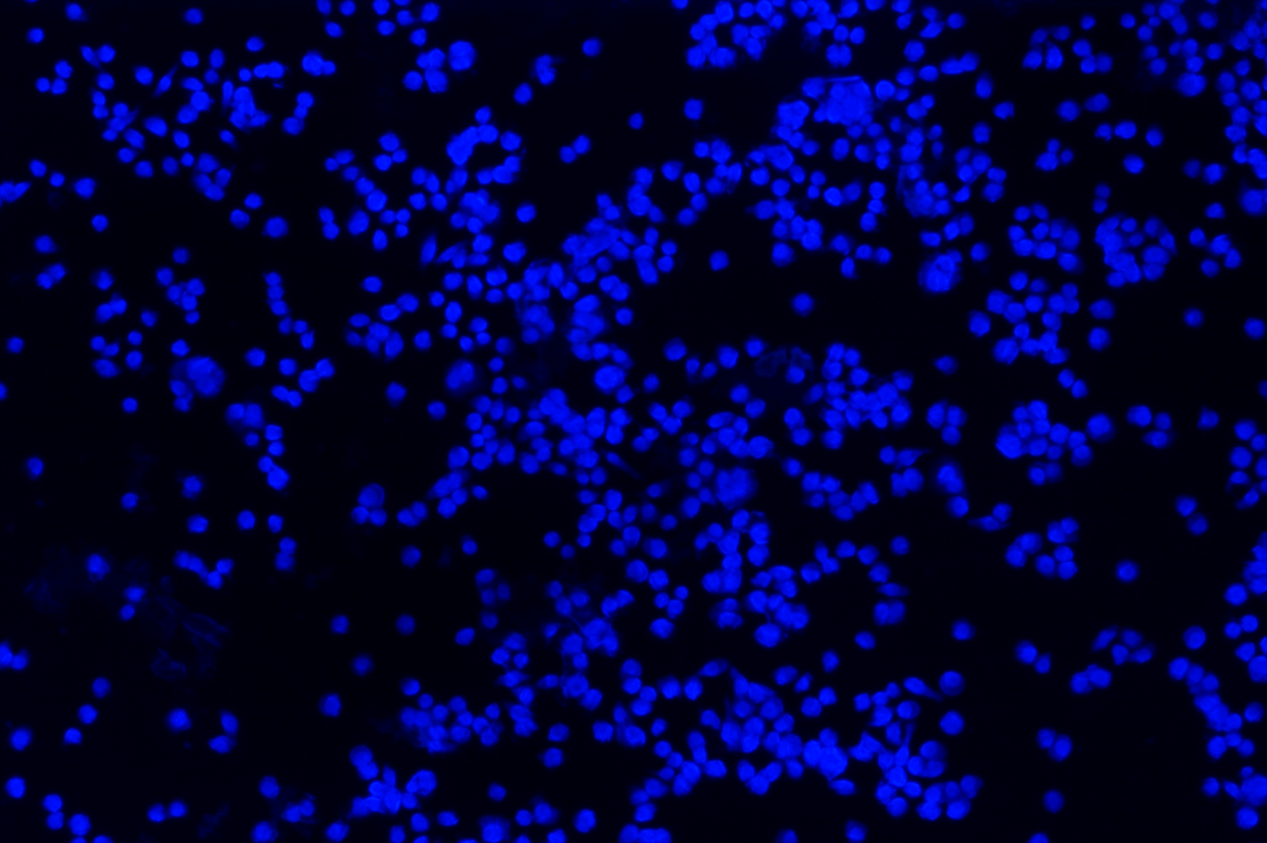
**

BGC-823-shPGM1-EdU

**
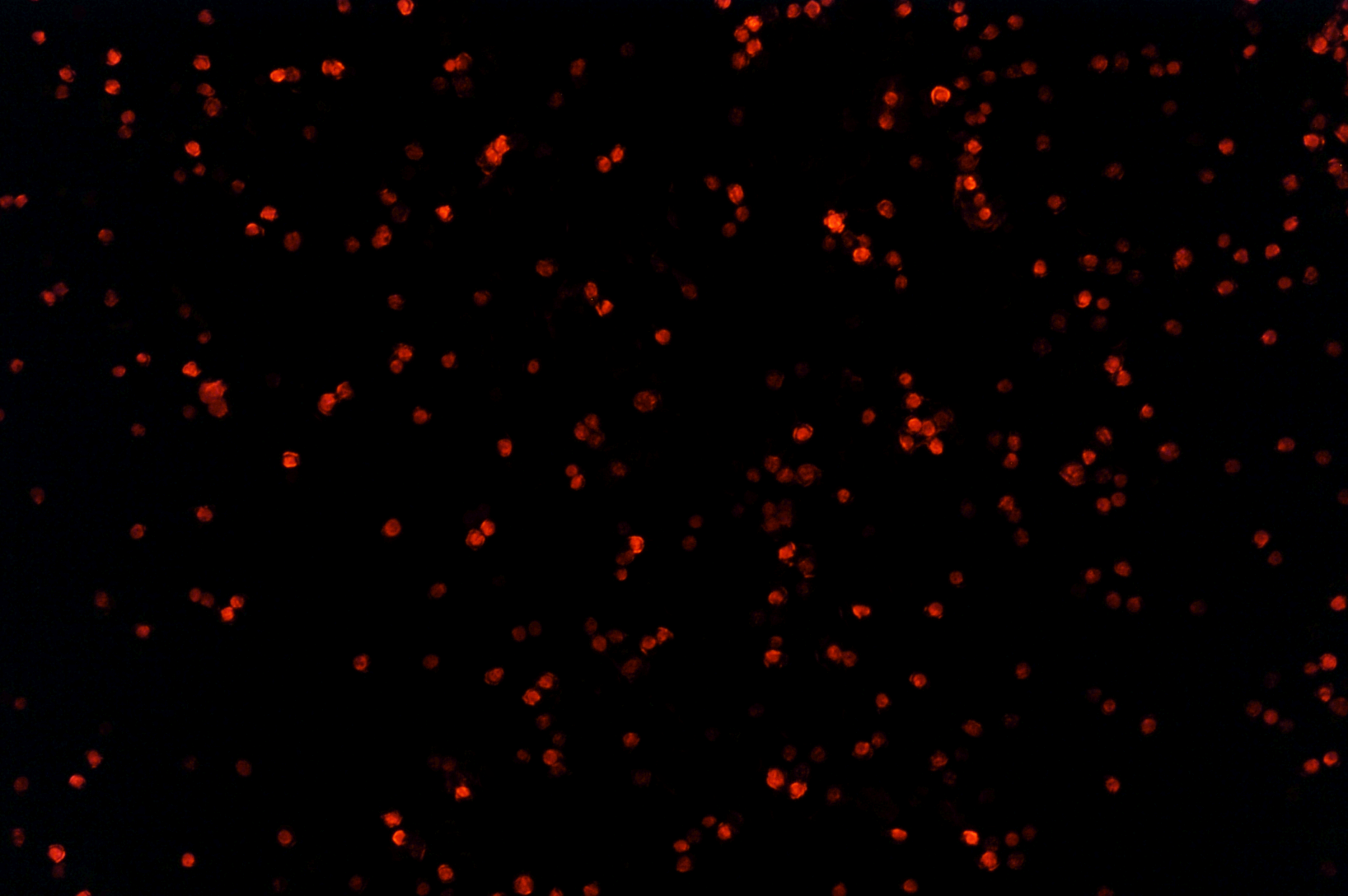
**

BGC-823- shPGM1+oePGM1-DAPI

**
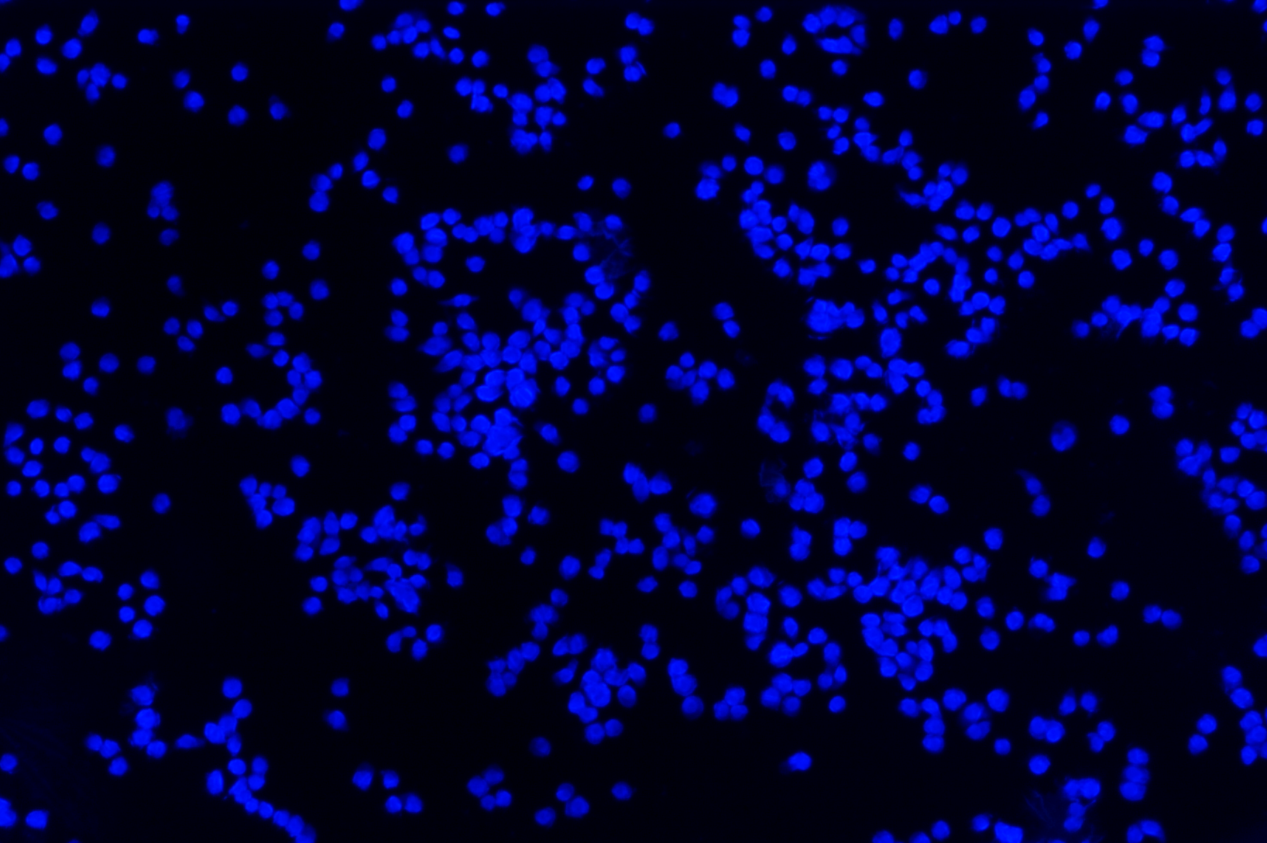
**

BGC-823- shPGM1+oePGM1-EdU

**
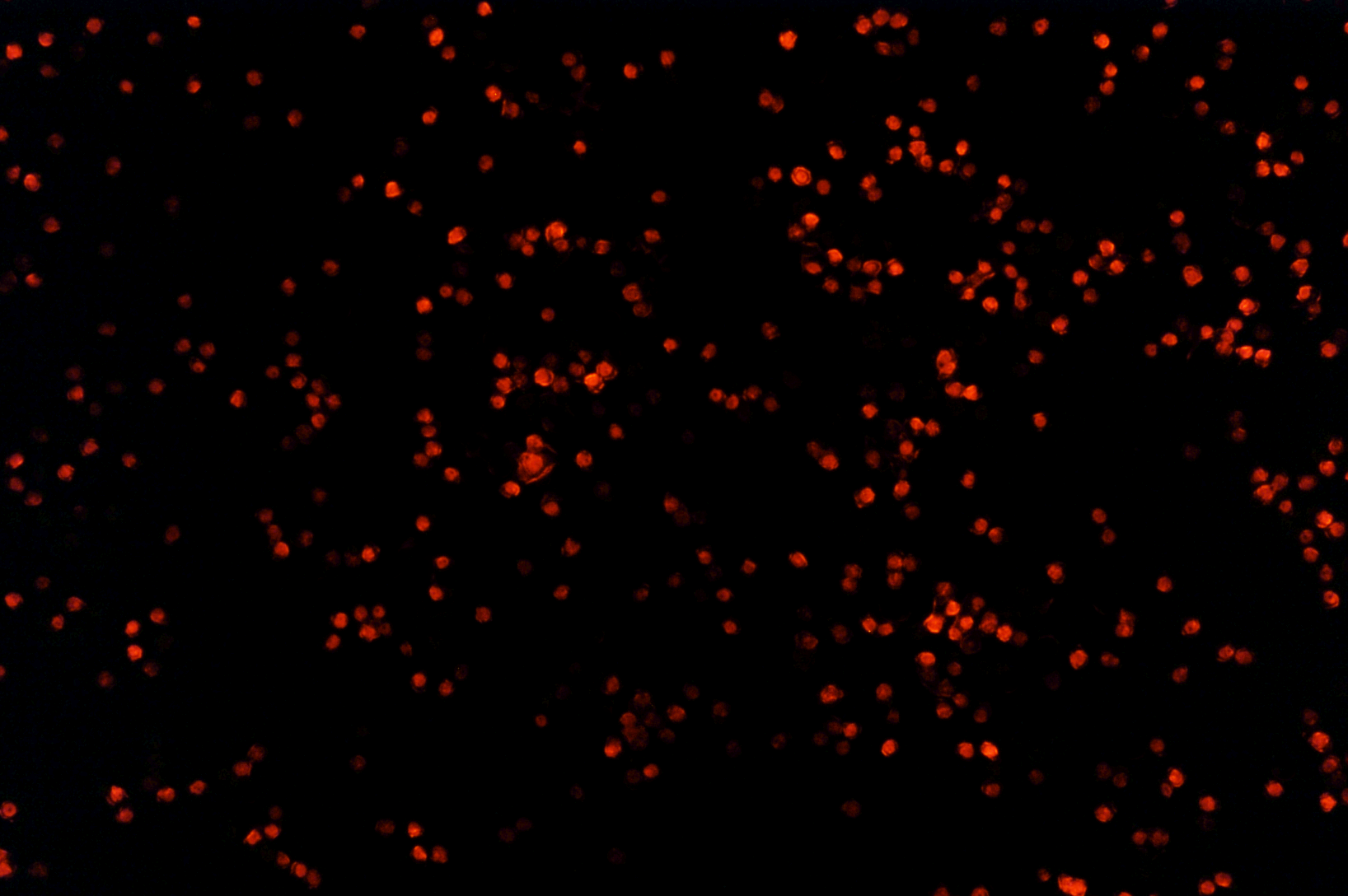
**

MKN-28-NC-DAPI


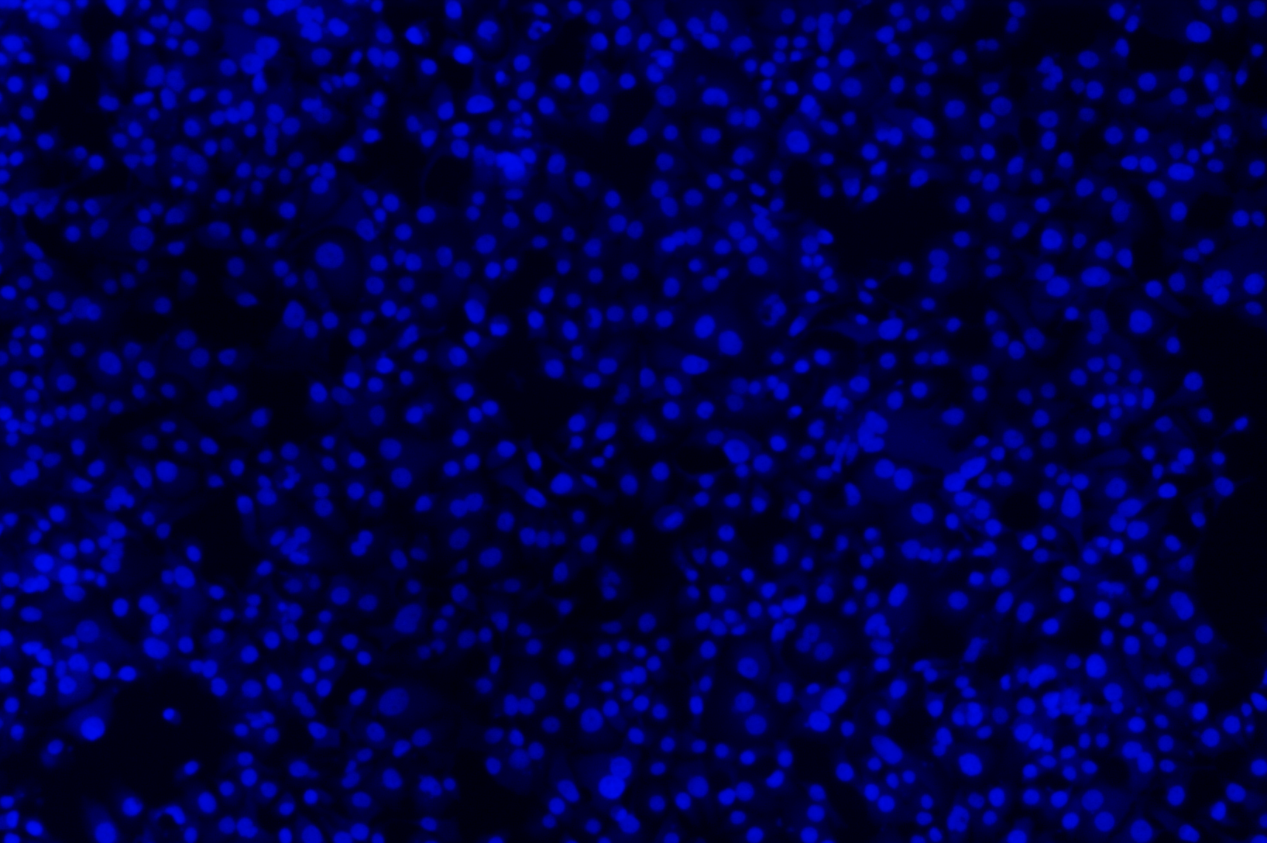


MKN-28-NC-EdU


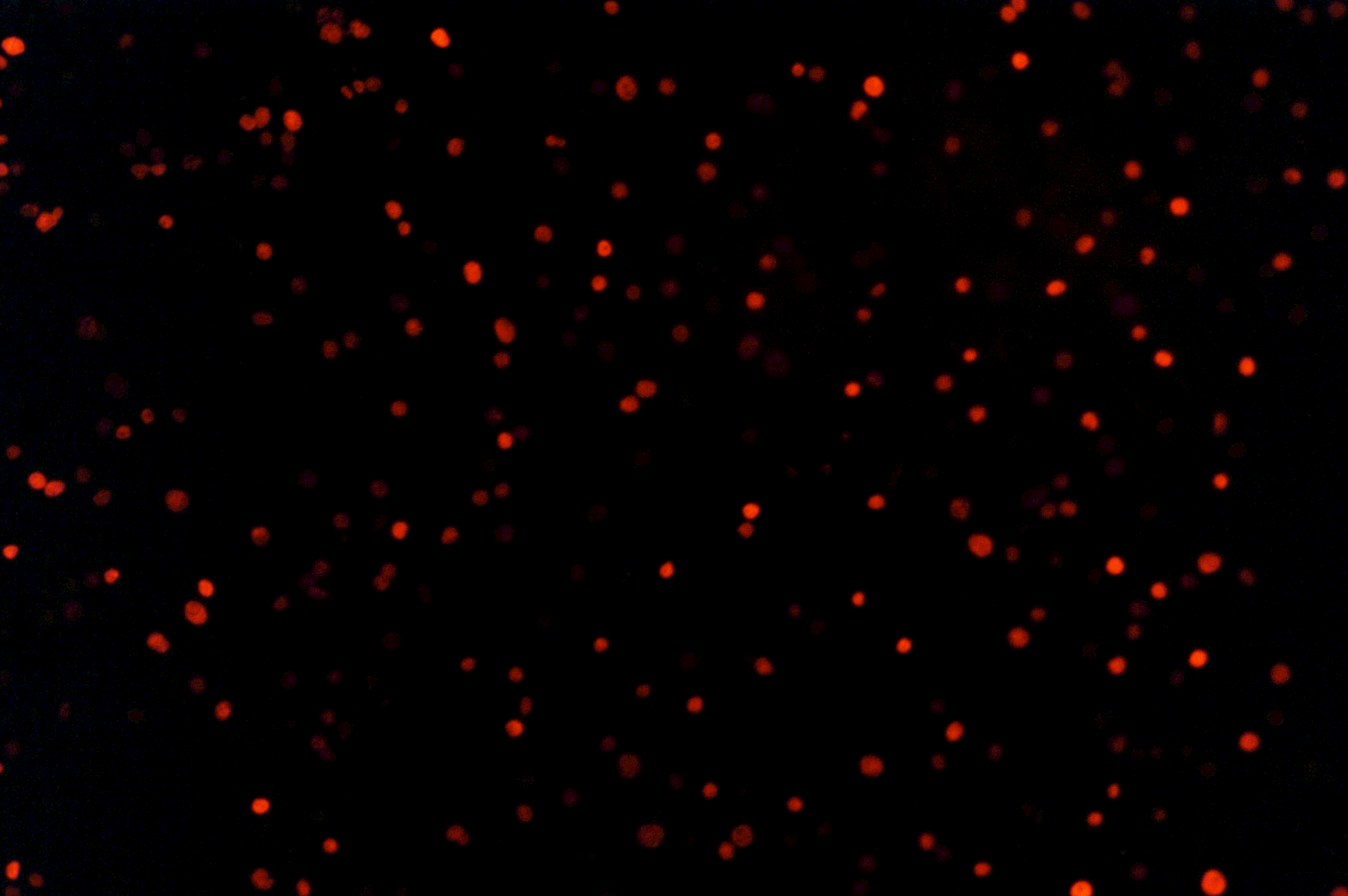


MKN-28- shPGM1-DAPI


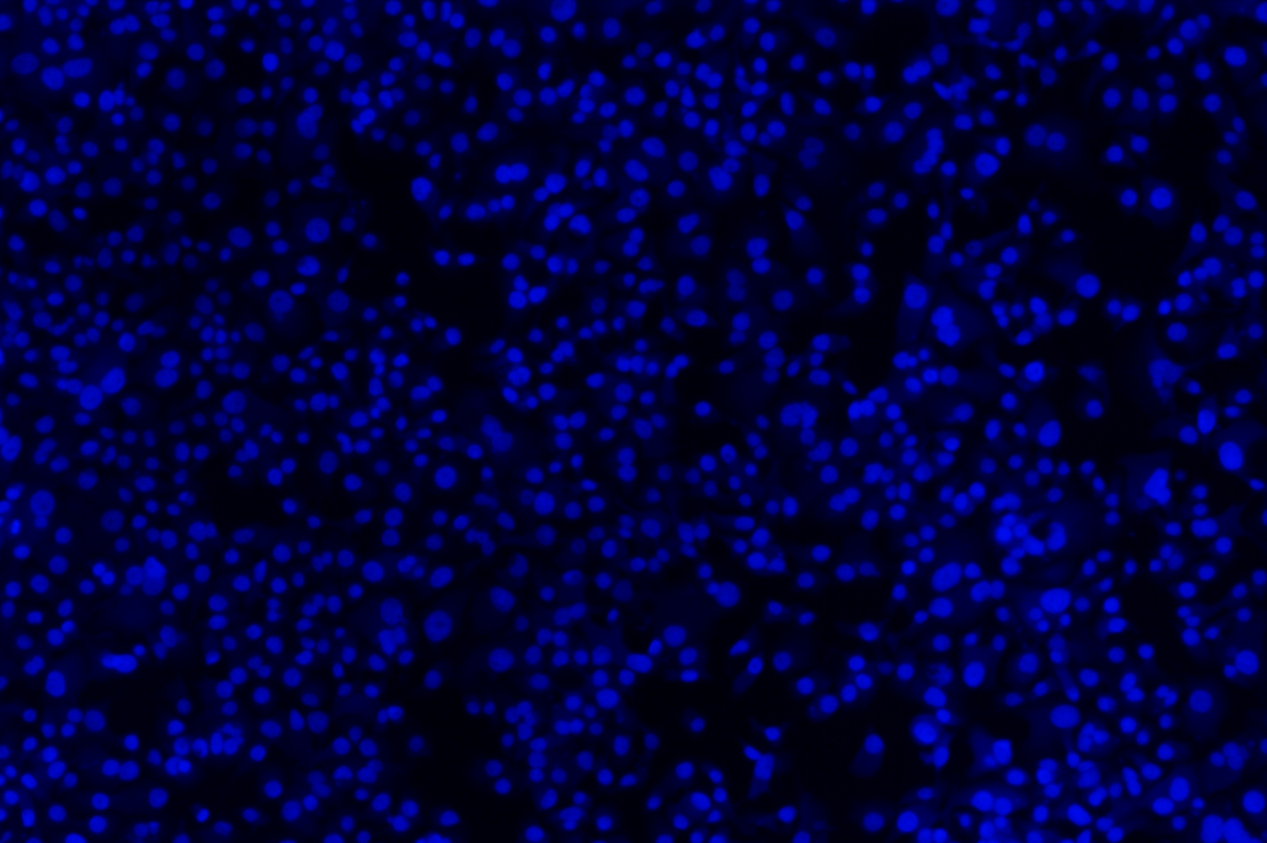


MKN-28-shPGM1-EdU


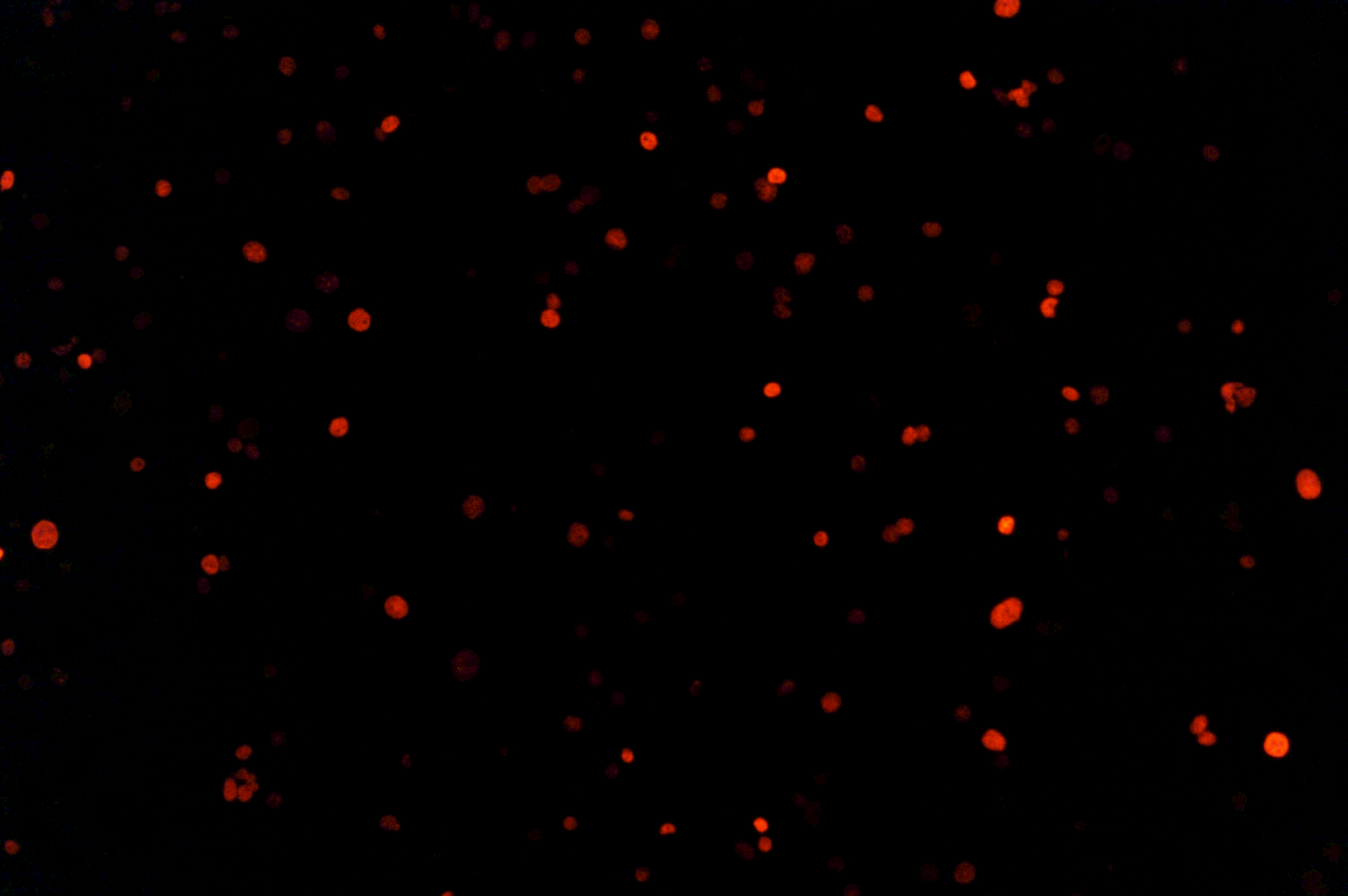


MKN-28- shPGM1+oePGM1-DAPI


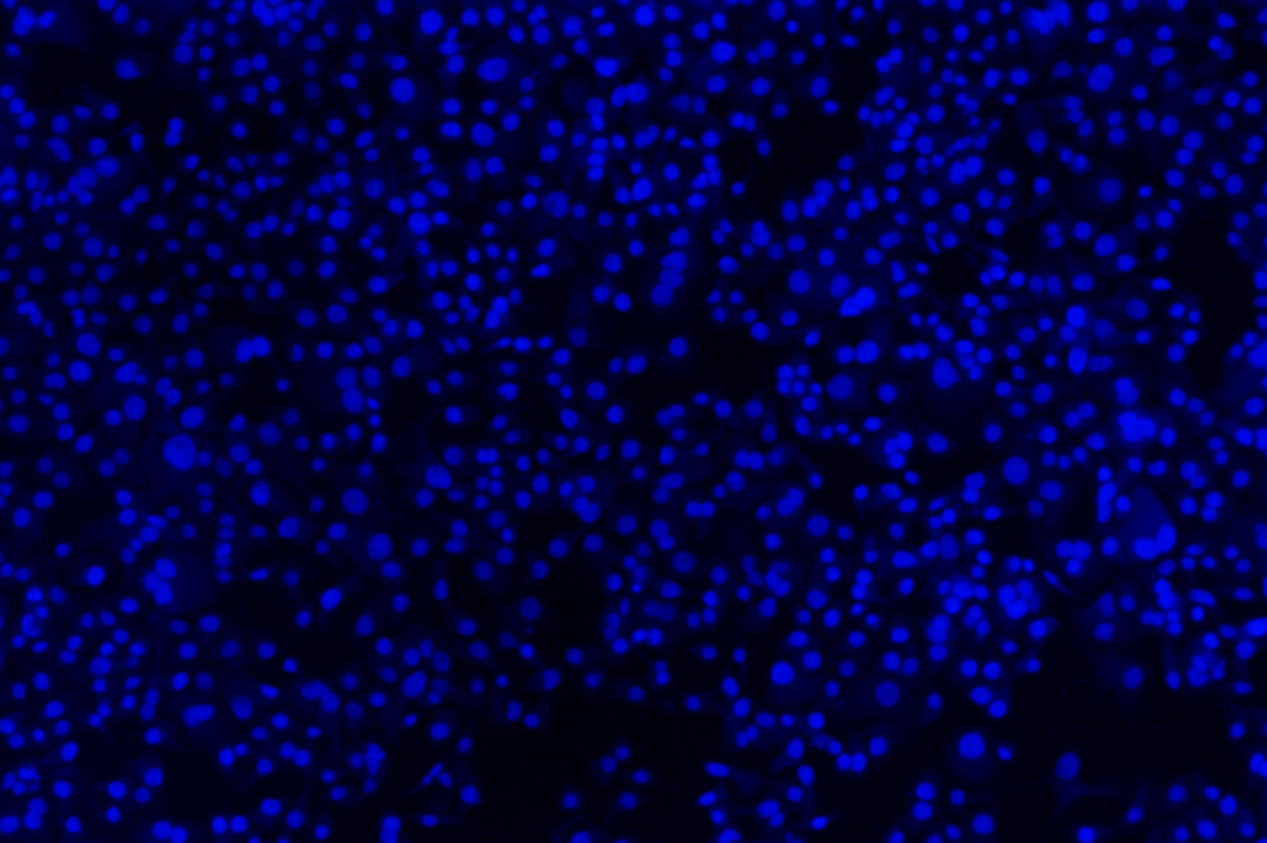


MKN-28- shPGM1+oePGM1-EdU


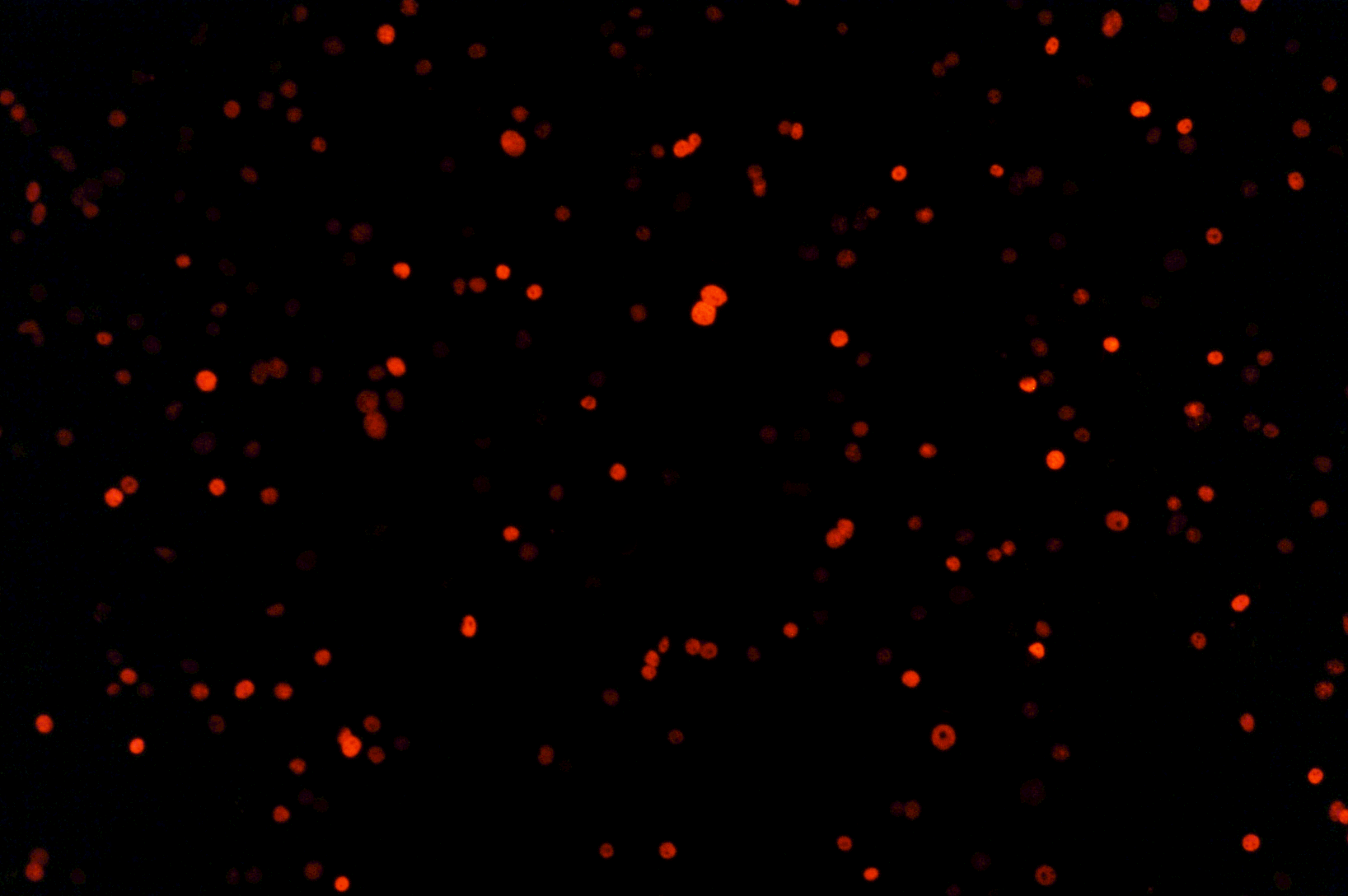


**Figure1-H-PGM1**


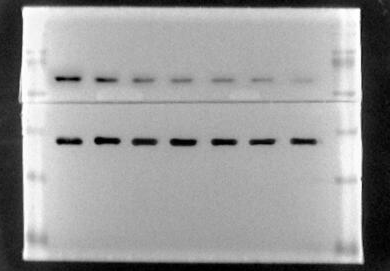


PGM1(~65)

β-actin(~43)

~35

~75

~63

~48

**Figure3-A-PGM1-BGC823**


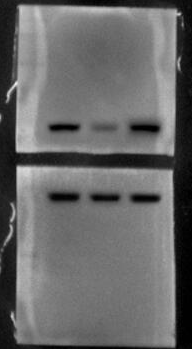


~35

β-actin(~43)

PGM1(~65)

~75

~48

~63

**Figure3-A-PGM1-MKN28**


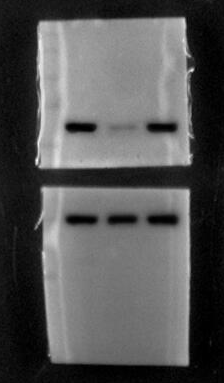


~75

~48

~35

~63

β-actin(~43)

PGM1(~65)

**Figure4-E-FASN-BGC823**


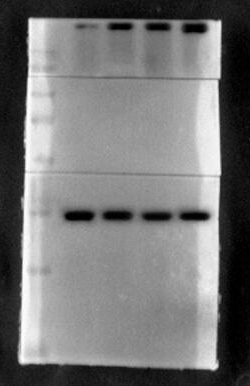


~63

~48

~35

FASN(~250)

β-actin(~43)

~180

**Figure4-E-FASN-MKN28**


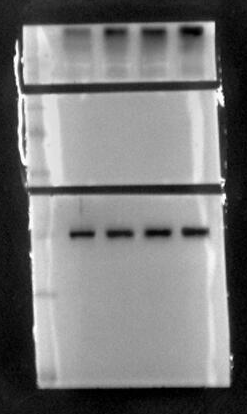


~180

~35

~63

~48

FASN(~250)

β-actin(~43)

**Figure4-E-CPT1A-BGC823**


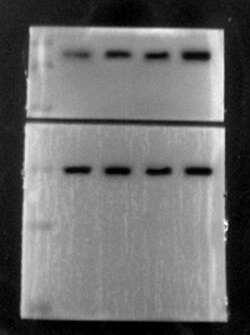


~48

CPT1A（~88）88

~100

~75

~63

~35

β-actin(~43)

**Figure4-E-CPT1A-MKN28**


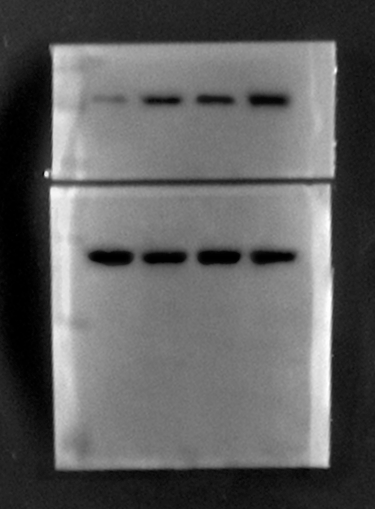


~35

~100

~75

~63

~48

CPT1A（~88）

β-actin(~43)

**Figure4-E-ACC-BGC823**


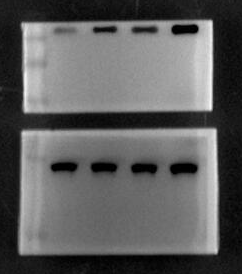


ACC(~265)

~25

~180

~48

β-actin(~43)

**Figure3-E-ACC-MKN28**


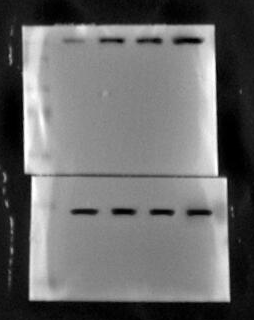


~180

~48

~25

β-actin(~43)

ACC(~265)

**Figure5-A-FASN-BGC823**


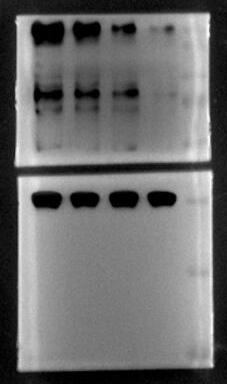


~48

~35

β-actin(~43)

~180

FASN(~250)

**Figure4-A-FASN-MKN28**


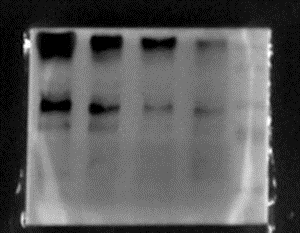


FASN(~250)

~180

**Figure5-A-CPT1A-BGC823**


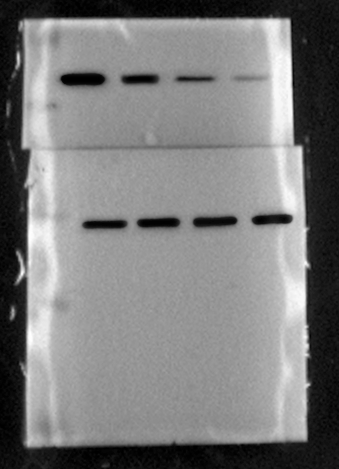


~100

~75

~35

~48

β-actin(~43)

CPT1A（~88）

**Figure5-CPT1A -MKN28**


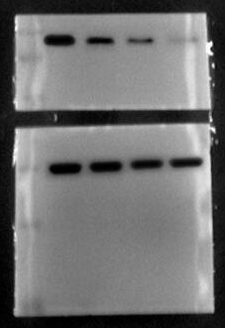


~100

~75

~48

~35

CPT1A（~88）

β-actin(~43)

~25

**Figure5-A-ACC-BGC823**


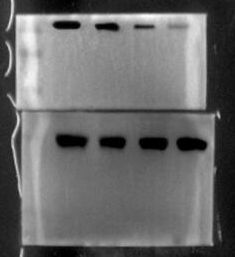


~180

~35

~48

~48

β-actin(~43)

ACC(~265)

**Figure4-A-ACC-MKN28**


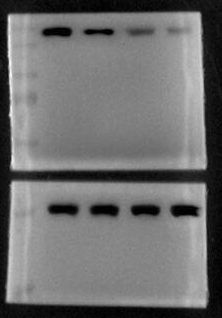


ACC(~265)

~180

~48

~25

β-actin(~43)
